# Gene expression and molecular pathway analyses differentiate immunotherapy-induced myositis from spontaneous dermatomyositis

**DOI:** 10.1038/s41598-025-11944-5

**Published:** 2025-08-04

**Authors:** Magdalena Röckel, Luca Musella, Corinna Preusse, Josefine Radke, Lisa Zimmer, Kai-Martin Thoms, Florentia Dimitriou, Matthias Endres, Wolfgang Böhmerle, Waltraud Fröhlich, Sami Tayb-Boulahfa, Sarah Leonard-Louis, Yves Allenbach, Carola Berking, Werner Stenzel, Samuel Knauss, Julio Vera, Lucie Heinzerling

**Affiliations:** 1 Department of Dermatology, Uniklinikum Erlangen, Erlangen, Germany; 2 Friedrich-Alexander-Universität Erlangen-Nürnberg (FAU), Erlangen, Germany; 3 Deutsches Zentrum Immuntherapie, DZI, Erlangen, Germany; 4 Charité - Universitätsmedizin Berlin, corporate member of Freie Universität Berlin and Humboldt-Universität zu Berlin, Department of Neuropathology, Charitéplatz 1, 10117 Berlin, Germany; 5 Charité - Universitätsmedizin Berlin, corporate member of Freie Universität Berlin and Humboldt-Universität zu Berlin, Department of Neurology, Charitéplatz 1, 10117 Berlin, Germany; 6 Department of Dermatology, Venerology and Allergology, Universitätsklinikum Essen, Essen, Germany; 7 University Medical Center Goettingen, Goettingen, Germany; 8 Department of Dermatology, University Hospital Zürich, Zürich, Switzerland; 9 Center for Stroke Research Berlin, Berlin, Germany; 10 Cluster of Excellence NeuroCure, Berlin, Germany; 11 German Center for Neurodegenerative Diseases (DZHE), Partner Site Berlin, Berlin, Germany; 12 German Center for Cardiovascular Research (DZHK), Partner Site Berlin, Berlin, Germany; 13 German Center for Mental Health (DZPG), Partner Site Berlin, Berlin, Germany; 14 Department of Internal Medicine and Clinical Immunology, Pitié-Salpêtrière University Hospital, Paris, France; 15 Department of Neuropathology, Sorbonne University Paris, Paris, France; 16 Department of Dermatology and Allergology, LMU University Hospital, Frauenlobstr. 9–11, 80337 Munich, Germany; 17 Side Effect Registry Immune-Oncology, SERIO, Munich, Germany; 18 Present Address: Institute of Pathology, University Medicine Greifswald, Greifswald, Germany

**Keywords:** Gene expression profiles, Immune Related adverse events, Immune checkpoint inhibitor therapy, Myositis, Anti-PD1/PD L1, Anti-CTLA-4, Cancer, Computational biology and bioinformatics, Genetics, Immunology, Molecular biology, Biomarkers, Diseases, Health care, Medical research, Molecular medicine, Neurology, Oncology

## Abstract

Immune checkpoint inhibitor therapy (ICI)-induced myositis (irMyositis) occurs in about 1% of patients treated with anti-PD1 or anti-CTLA-4 antibodies and can be debilitating or even fatal. We compared gene expression profiles from skeletal muscle biopsies between irMyositis patients, patients with spontaneous dermatomyositis (DM, comprising anti-Mi2-positive and anti-TIF1-γ-positive subtypes), and non-diseased controls (NDC). We used the NanoString nCounter PanCancer Immune Profiling Panel to perform differential gene expression (DGE) and pathway enrichment analyses. We identified 93 differentially expressed genes (DEGs) across conditions. Gene set enrichment analysis (GSEA) suggested activation of interferon gamma (type-II IFN) and interferon alpha/beta (type-I IFN) signaling in irMyositis and DM, respectively. For instance, type-II IFN was upregulated exclusively in irMyositis when compared to DM, which conversely showed upregulation of effector genes downstream type-I IFN. The observed fold-change of a subset of 33 genes drove the GSEA. We further characterized the DEGs using network interaction and expression correlation analyses. This allowed us to describe potential differences between regulatory hubs and cells involved in irMyositis susceptible to ICI effects. For example, the downregulation of *FOXP3* we observed together with the upregulation of the chemokine *CCL14* in irMyositis may have been a consequence of T cell activation upon ICI therapy. The gene expression correlation and putative molecular interactions set irMyositis apart from DM, particularly with respect to IFN response and DGE of interactors of ICI protein targets (CTLA4, PD-1, PD-L1). Our results suggest new avenues for understanding immunotherapy-related adverse events.

**Supplementary Information:**

The online version contains supplementary material available at 10.1038/s41598-025-11944-5.

## Introduction

Immune checkpoint inhibitor (ICI) therapy can induce a plethora of immune-related adverse events (irAE). While immune-related colitis, hepatitis and endocrine side effects occur frequently, neuromuscular side effects are rare with a frequency of less than 3%^1^. IrMyositis is the most frequent neuromuscular irAE occurring in up to 1% of patients treated with anti-PD1 and/or anti-CTLA-4 antibodies^2^. IrMyositis can be complicated by concomitant myocarditis (irMyocarditis) or Myasthenia-gravis-like symptoms, which often leads to long term sequelae^3^, disability or even fatalities^4^. Importantly, one in two patients documented with irMyositis/irMyocarditis died in a large adjuvant clinical trial^5^. Thus, diagnostic and therapeutic approaches must be improved.

So far, irMyositis has often been treated in analogy to spontaneous autoimmune dermatomyositis despite important clinical and immunological differences between the ICI-induced and idiopathic inflammatory myopathies (IIMs)^2,6^. Myositis-associated autoantibodies including autoantibodies against nuclear and cytoplasmic antigens are rarely detected in irMyositis, whereas they are positive in up to 89% of patients with IIM^7–9^. Dermatomyositis is considered a highly heterogeneous entity, with six main groups, namely anti-TIF1-γ, anti-Mi2, anti-MDA5, anti-NXP2, anti-SAE, and autoantibody negative subtypes^10–12^. It can be difficult to distinguish between irMyositis and IIMs associated with malignancy but cutaneous features like Gottron sign, heliotrope rash or periungual erythema typically present in anti-Mi2 dermatomyositis (Mi2 subtype) can help to determine the diagnosis. Histologically, irMyositis can manifest itself by marked signs of necrosis, perifascicular atrophy and vascular damage, or pro-inflammatory cell infiltrates^13^. Mi2 subtype patients respond well to standard treatment, including corticosteroids and rituximab or other autoantibody reducing approaches (plasmapheresis) and show a good overall prognosis^14–17^ while anti-TIF1-γ dermatomyositis (TIF1γ subtype) is more therapy-refractory. As with IIM, common symptoms of irMyositis patients are myalgia and progressive, proximal upper and lower extremity weakness. Additionally, symptoms such as myopathy of external ocular muscles and diplopia can resemble those of myasthenia gravis. However, compared to myasthenia gravis, irMyositis shows a more sudden onset and no fluctuation of symptoms or fatigability^18^. Elevation of creatine kinase levels, myopathic changes in the electromyogram and necrotic myofibers in muscle biopsies are found in patients diagnosed with irMyositis^18^.

In this study, we measured gene expression at the RNA level to elucidate the molecular mechanisms involved in irMyositis and DM, to better understand similarities and differences in pathological mechanisms that could guide diagnosis and treatment. Using the NanoString nCounter PanCancer Immune Profiling Panel^19^, we determined the expression levels of 770 genes related to immune-oncological signaling pathways and cell types and identified a set of significantly differentially regulated genes from muscle biopsies. Most notably, tissue extracts of irMyositis patient biopsies showed distinct immunological pathways from those activated in anti-Mi2-positive and anti-TIF1-γ-positive ones, which may suggest novel therapeutic research strategies.

## Methods

### Patient cohorts

**Fig. 1:**
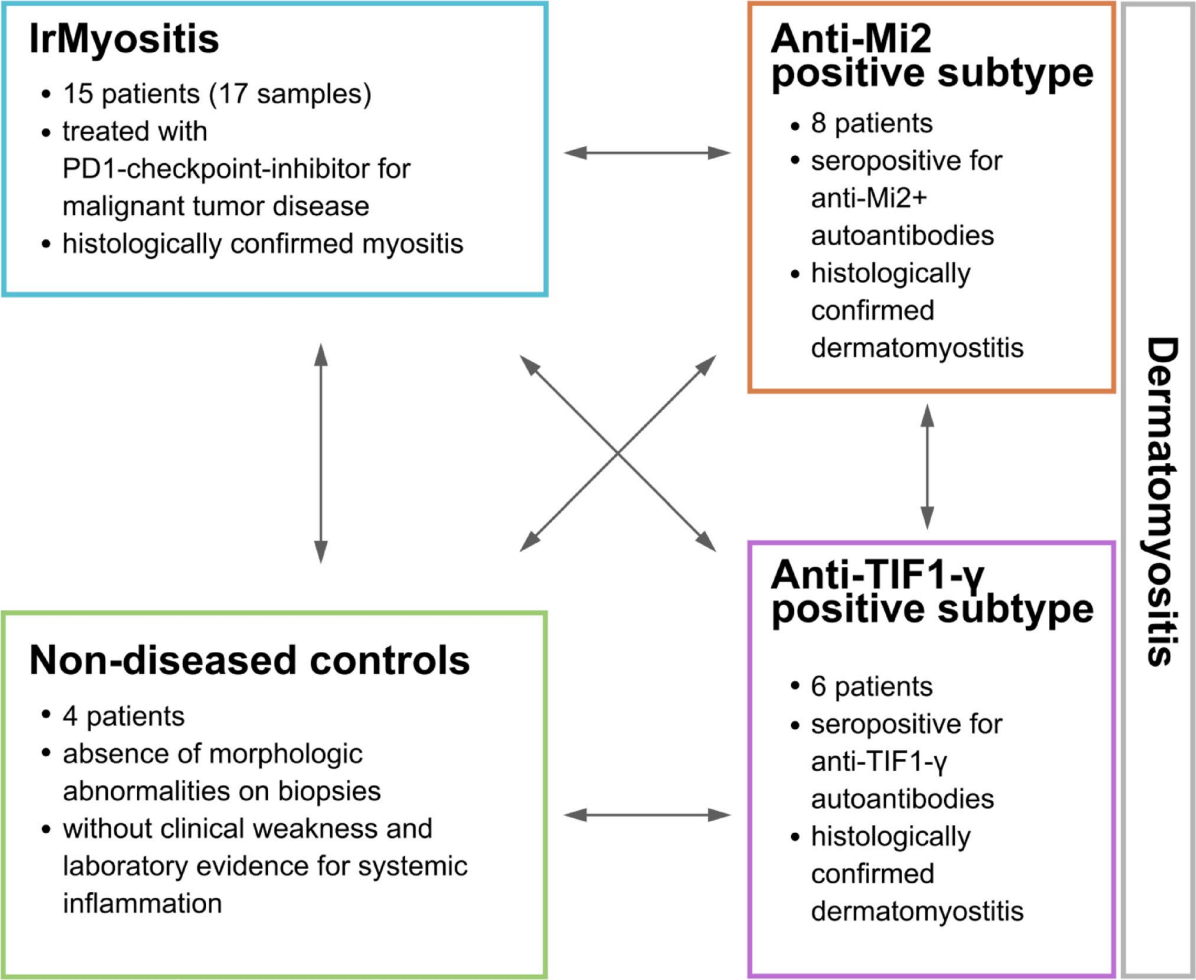
Schematic representation of patient registry (33 patients) and concept of analysis. The patient cohort is subdivided into irMyositis, Mi2 subtype and TIF1γ subtype, and NDC. Arrows indicate the comparisons investigated in this study.

The study design and concept of analysis is described in Fig. 1. Skeletal muscle biopsies were analyzed from patients diagnosed with irMyositis (n = 15, 17 samples), Mi2 subtype (n = 8, 8 samples), and TIF1γ subtype (n = 6, 6 samples) DM, from Berlin, Erlangen, Essen, Paris, and Zurich. The diagnosis of irMyositis was deemed likely when symptoms occurred in close-time association to ICI therapy initiation and if other causes were excluded. All cases of irMyositis, except for two, were diagnosed in patients who had been treated for cutaneous melanoma. Additionally, we investigated skeletal muscle biopsies from patients with nonspecific complaints in the context of “fatigue-like” symptoms, without clinical muscle weakness, absence of morphologic abnormalities on skeletal muscle biopsies, absence of elevated creatine kinase (CK) levels or laboratory evidence of any systemic inflammation, serving as NDC (n = 4, 4 samples). Table 1 presents the demographics and clinical characteristics of patients included in the analysis. We derived the collected data from clinical health records submitted after patient consent to the digital hospital information systems in the participating centers in Berlin, Erlangen, Essen, Göttingen, Paris and Zurich. It includes muscle strength assessments, CK levels, autoantibody profiles, and characteristics of muscle pathology. CK levels were within range in the NDC group and significantly elevated in the diseased groups. All muscle biopsy samples analyzed in this study were obtained from patients between 22 and 86 years of age. The analysis included samples from 17 female and 18 male patients. The subgroup of NDC includes an equal number of male and female participants. In the Mi2 and TIF1γ subtype group female patients predominate, whereas male participants are predominant in the irMyositis group. As most patients suffered from cutaneous melanoma, the employed ICI therapies were anti-PD-1 (nivolumab or pembrolizumab), in some patients in combination with anti-CTLA-4 (ipilimumab). This study was approved by the institutional review boards at the Friedrich-Alexander University Erlangen-Nürnberg (2_20B), the LMU University Hospital Munich (20–1122) and at the Charité –Universitätsmedizin Berlin (EA2/163/17). All research was performed in accordance with the legally binding regulations in Germany and the Declaration of Helsinki for research involving human participants. We obtained informed consent from all participants and/or their legal guardians before handling samples and processing data.

**Table 1:** Clinical data documentation (n = 33, 35 samples). Demographics and clinical characteristics of patients, obtained from clinical health records submitted to digital hospital information systems in the participating centers in Berlin, Erlangen, Essen, Göttingen, Paris and Zurich. Patient ID from which multiple biopsies have been sampled are indicated with a second numbering system (e.g. “19.1”). For improved readability, patient characteristics have been abbreviated: NDC (A), Mi2 subtype (B), TIF1γ subtype (C), and irMyositis (D) groups; monotherapy with Pembrolizumab (Pembro), Nivolumab (Nivo) or Ipilimumab (Ipi), as well as a combination approach of Ipilimumab/Nivolumab (Com.); muscle pathology characteristics typically found in dermatomyositis (DMS.) or necrotizing myopathy (N.M.); patient information not reported by the center or data is not part of routine patient assessment (N.R.); not-applicable or unperformed treatment or diagnostic approach (N/A); no treatment (steroids or immunosuppressants) at time of biopsy (N.T.); no autoantibody found (Neg.); age at time of biopsy (Age); body weight (b.w.); methylprednisolone (mp.); ophthalmoplegia (op.); tetraparesis (tp.); hemiplegia (hp.); axial weakness (aw.); cutaneous Melanoma stage IV (C.M. IV) and Cutaneous melanoma stage not reported (C.M. S.N.R.); endometrioid adenocarcinoma (E.A.); skin lesions (S.L.); bulbar involvement (B.I.). Further commentaries are abbreviated by #: steroids, IVIG, plasmapheresis, hemodialysis, intensive care treatment; ##: left hip flexor palsy M4 and hyposensitivity ventral thigh; ###: MGUS/metastatic melanoma (subcutaneous and soft tissue metastases).

Group	Patient ID	Muscle Pathology Characteristics	Autoantibody Profiles	Type of ICI Therapy	Treatment at Time of Biopsy	Sex (M/F)	Age	Muscle Strength Assessments	Ck Level (U/I)	Comorbidities/Malignancy	Extramuscular Manifestations
A	1	normal	N.R.	N/A	N.R.	F	39	N.R.	normal	N.R.	N.R.
	2	normal	N.R.	N/A	N.R.	M	64	N.R.	normal	N.R.	N.R.
	3	normal	Neg.	N/A	N.T.	M	22	normal	127	none	none
	4	normal	Neg.	N/A	N.T.	F	49	normal	normal	none	none
B	5	DMS.	anti-Mi-2	N/A	N.R.	F	86	proximal tp.	N.R.	N.R.	S.L.
	6	DMS.	anti-Mi-2	N/A	N.R.	F	83	proximal tp.	9,000	N.R.	S.L.
	7	DMS.	anti-Mi-2	N/A	N.T.	M	54	4/5 diplegia (arms)	N.R.	none	S.L.
	8	DMS.	anti-Mi-2	N/A	N.R.	F	82	4/5 tp.	N.R.	none	S.L.
	9	DMS.	anti-Mi-2	N/A	N.R.	F	62	4/5 tp.	6,400	none	S.L.
	10	DMS.	anti-Mi-2	N/A	N.T.	F	48	N.R.	N.R.	none	S.L.
	11	DMS.	anti-Mi-2	N/A	N.T.	M	57	4/5 proximal hp	N.R.	none	S.L.
	12	DMS.	anti-Mi-2	N/A	N.T.	F	86	4/5 proximal hp	N.R.	N.R.	S.L.
C	13	DMS.	anti-TIF-1γ	N/A	N.T.	F	62	3–4/5 tp.	N.R.	breast cancer	S.L., B.I.
	14	DMS.	anti-TIF-1γ	N/A	N.T.	M	71	4/5 tp.	2,000	none	S.L.
	15	DMS.	anti-TIF-1γ	N/A	N.T.	F	60	4/5 tp.	2,400	E.A.	S.L.
	16	DMS.	anti-TIF-1γ	N/A	N.T.	F	48	4/5 tp.	N.R.	N.R.	none
	17	DMS.	anti-TIF-1γ	N/A	N.T.	F	69	4/5 tp.	11,500	N.R.	none
	18	DMS	anti-TIF-1γ	N/A	N.T.	F	64	N.R.	N.R.	none	skin calcinosis
D	19.1	N.M.	Neg.	Com.	N.R.	M	75	proximal tp.	N.R.	C.M. S.N.R.	N.R.
	19.2	N.M.	Neg.	Com.	N.R.	M	75	proximal tp.	N.R.	C.M. S.N.R.	N.R.
	20	signs of inflammation	Neg.	Pembro	steroids	M	75	multiple^##^	6,870	multiple^###^	myocarditis
	21	N.R.	Neg.	Pembro	prednisolone 0.5 mg/kg b.w	M	75	N/A	1,626	C.M. IV	transaminitis, myocarditis
	22	N.R.	N/A	Com.	mp. 1 mg/kg b.w	M	69	N/A	301	C.M. IV	hepatitis G3, thyreoiditis
	23.1	lymphocytic infiltrates	anti-cN-1A	Ipi, Pembro	steroids	M	58	proximal hp.	2,400	C.M. IV	none
	23.2	lymphocytic infiltrates	anti-cN-1A	Ipi, Pembro	steroids	M	58	proximal hp.	2,400	C.M. IV	none
	24	N.M.	Neg.	Pembro	mp. 1000 mg	M	61	5/5 peripheral extremities, aw., op.	11,900	C.M. S.N.R.	vitiligo
	25	N.M.	Neg.	Nivo	mp. 500 mg	M	74	upper legs and aw., diplopia	6,200	lung cancer	B.I.
	26	N.M.	Neg.	Nivo/anti-TIGIT	N.T.	F	63	4/5 hp.	2,705	renal cancer	B.I., dyspnea
	27	N.M.	Neg.	Pembro	N.T.	F	86	aw., op., ptosis	1,400	C.M. S.N.R.	ptosis
	28	N.M.	Neg.	Pembro, Nivo	N.T.	M	75	aw.	N.R.	C.M. S.N.R.	none
	29	N.M.	Neg.	Nivo	N.T.	F	76	tp.	N.R.	C.M. S.N.R.	none
	30	N.M.	Neg.	Nivo	N.T.	M	83	upper legs and aw.	1,059	C.M. S.N.R.	none
	31	signs of atrophy and inflammation	Neg.	Com.	steroids	F	59	N.R	625	C.M. IV	colitis, arthritis
	32	N.M.	Neg.	Com.	multiple^#^	M	77	N.R	> 42,670	C.M. S.N.R.	none
	33	N.M.	Neg.	Nivo	N.R.	M	84	tp.	N.R.	C.M. S.N.R.	N.R.

### RNA isolation

We isolated total RNA from muscle specimen using TRIzol Reagent (Thermo Fisher Scientific, Germany), as previously described^20^.

### NanoString-based sample profiling

For each sample, we quantified gene expression using the NanoString nCounter PanCancer Immune Profiling Panel (NanoString, XT-CSO-HIP1-12). The panel consists of six positive control probes, eight negative control probes, and 770 probes aimed at detecting the RNA levels of that many *H. sapiens* genes. 40 out of the 770 probes are specific for a set of housekeeping genes. The rest of the probes account for a set of well-known cancer and immune response-associated genes. After checking that RNA quality was adequate, we used 200–500 ng of total RNA as input and performed sample hybridization according to the manufacturer’s instructions. We completed the data acquisition on an nCounter Digital Analyzer (NanoString, Seattle, USA).

### NanoString data processing and normalization

We extracted count data from NanoString RCC files using the *nanostringr* library (version 0.41) for the R programming language (version 4.2.3)^21,22^. For each sample, we evaluated the quality of the raw counts for each gene-probe hybridization by comparing them with the average counts of negative control probes. After normalizing by the sample library size, if a gene had corresponding counts-per-million (CPM) higher than the average negative control count plus two standard deviations in less than four samples (the size of NDC subtype, the smallest in the cohort), we removed that gene from the analysis. The rationale was to guarantee that all analyzed genes had adequate expression in most samples, to avoid statistical imbalances^23^. Next, following NanoString guidelines, we scaled the raw counts of the remainder genes across samples using CodeSet Content Normalization; briefly, sample-specific scaling factors are computed based on the ratio between the average geometric mean of housekeeping gene counts and the geometric mean of the whole-sample counts (see MAN-C0011-04, available at https://university.nanostring.com, for further details).

### Statistical analysis and differential gene expression

Similarly to previous work on NanoString data, we employed the *limma-voom* framework to model the mean–variance relationship of log-CPM normalized data^24–27^. We further compensated for the within-sample expression variation using the trimmed mean of M-values (TMM) normalization on CPM values upon applying *voom*. Then, we constructed a linear model of gene expression using disease subtype (irMyositis, Mi2 subtype, TIF1γ subtype, and NDC), cartridge (batch), and biopsy conservation (FFPE, cryopreservation) as explanatory variables (covariates). We additionally modeled patient variation as a random effect to account for correlation between sample biopsies derived from the same patient, using the *limma* function *duplicateCorrelation* as described previously^27^. After correcting for the unwanted covariate effects, we carried out DGE analysis across disease subtypes via empirical Bayes moderation of standard errors (pairwise, moderated t-test and analysis of variance of the corresponding t-statistics via F-statistics). P-values resulting from F-tests were adjusted to 5% FDR using Benjamini–Hochberg correction. To correctly account for multiple testing both within and across pairwise comparisons (post hoc t-test), we selected the *hierarchical* modality within the *limma* function *DecideTests*; briefly, this adjustment method first computes a scaling factor based on the number of significant F-tests within the nominal FDR (5%), then it accordingly recomputes the FDR level, and finally adjusts t-tests comparison-wise.

### Enrichment analysis

We used the observed gene-wise fold-changes to perform GSEA using the R library *ClusterProfiler*^28^. As gene sets, we used Reactome pathways with sizes ranging from 3 to 100 genes.

### Network reconstruction and representation

We reconstructed a gene network of directed molecular interactions by accessing the Reactome graph database using Neo4j and CYPHER queries^29^. We used the *SchemaClass* of *Regulation* nodes to curate the mode of regulatory interactions. We used the cellular component annotation in *EntityWithAccessionedSequence* nodes to curate the subcellular or extracellular localizations of the interacting genes. To prune the Reactome network, we computed all pairs, directed shortest paths (APSPs) between a subset of DEGs, by means of *igraph* implementation of Dijkstra’s algorithm^30^.

### Expression correlation tests

We used Spearman’s correlation to measure the strength of association between the expression levels of gene pairs across the sample cohort. We applied asymptotic *t* distribution and Edgeworth series approximations to perform the correlation test statistics. After Benjamini–Hochberg correction, correlations were considered significant at a nominal FDR of 5%. We curated immune-cell-specific biomarkers using the whitepaper of the nCounter PanCancer Immune Profiling Panel (NanoString, XT-CSO-HIP1-12)^31^.

### Result validation using deposited RNAseq data and literature mining

To compare DGE results of this study to previously published expression data, we downloaded and re-analyzed from Gene Expression Omnibus the RNAseq dataset entry GSE220915^32^. The dataset consists of RNA extracts from muscle biopsies classified as dermatomyositis, comprising anti-Mi2 (n = 12), anti-NXP2 (n = 14), anti-TIF1γ (n = 12), and anti-MDA5 (n = 6) subtypes, antisynthetase syndrome (n = 18, patients tested positive for anti-Jo1 autoantibodies), immune-mediated necrotizing myopathy (n = 54, patients tested positive for anti-SRP or anti-HMGCR autoantibodies), inclusion body myositis (n = 16), and histologically normal muscle biopsies (n = 33). After normalizing by the sample library size, if a gene had corresponding CPM higher than 10 in less than 16 samples, we removed that gene from the analysis. Information on batches or other covariates was not available, and we therefore formulated the linear model regression only in terms of sample classification. We conducted DGE analysis in the same fashion as with the NanoString dataset using *edgeR* and *limma*, except for CodeSet Content Normalization*.* See **Supp. Information** for code implementation.

We conducted a comprehensive, semi-automatic review of biomedical literature concerning irAE, ICI therapy especially in melanoma, and DM using ENQUIRE (software implementation deposited as apptainer image at 10.6084/m9.figshare.29357207.v1)^33^. First, we formulated the following PubMed queries: (i) ((("immune checkpoint inhibitor*"[MeSH Terms])) AND (“autoimmune diseases”[MeSH Terms])) NOT (“review”[Publication Type]), “10 years” and “Abstract” filters, 239 results as of 17.06.2025; (ii) *(("immune checkpoint inhibitors/adverse effects"[MeSH Terms] AND “melanoma”[MeSH Terms]) OR ("dermatomyositis/immunology"[MeSH Terms])) NOT (“review”[Publication Type])*, “10 years” and “Abstract” filters, 660 results as of 17.06.2025. Using the resulting list of PMIDs as input, we generated a network of significantly co-occurring genes and MeSH terms and corresponding source literature via ENQUIRE. The network was then converted to a graph database using Neo4j and interrogated via CYPHER queries. See **Supp. Information** for code implementation.

### Statistical software

Table 2 and Supp. Information report the software we used to conduct the data analysis.

**Table 2:** R Functions and parameters used for NanoString data analysis. See Methods for in-depth description of the procedures.

Procedure	Code snippets (pseudocode)	Library/Database (version)
RCC file import	counts = parse_counts(RCC file), attributes = parse_attributes(RCC file)	*nanostringr* (0.1.0)
CPM normalization	calcNormFactors(DGEList object, method = “none”)	*edgeR* (3.40.2)
Filtering of lowly expressed genes	filterByExpr(DGEList-object, group = disease subtype, min.count = 2^(mean(per-probe average negative-control count) + 2 * sd(per-probe average negative-control count)))	*edgeR* (3.40.2)
CodeSet Content Normalization	in-house script (Supp. Information)	R *base* (4.2.3)
Design matrix creation	model.matrix(~ 0 + disease subtype + cartridge + conservation)	R *stats* (4.2.3)
Heteroschedasticity correction (*voom*)	voom(calcNormFactors(DGEList object, method = “TMM”), design = model.matrix(…), normalize.method = “none”, plot = F)	*limma* (3.54.0)
Intersample correlation estimation	duplicateCorrelation(voom output, model.matrix(…), block = Patient-ID)$consensus.correlation	*limma* (3.54.0)
Linear model regression	lmFit(voom output, model.matrix(…))	*limma* (3.54.0)
Contrast matrix creation	in-house script (Supp. Information)	R *base* (4.2.3)
Fold-change estimation and statistical inference (moderated t-test, F-test)	contrasts.fit(lmFit output, contrast matrix); eBayes(contrasts.fit(…),trend = F,robust = F)	*limma* (3.54.0)
Statistical inference (post hoc moderated t-test)	decideTests(eBayes output,method = ‘hierarchical’, adjust.method = ‘BH’, p.value = .05))	*limma* (3.54.0)
APSPs identification	all_shortest_paths(Reactome graph, from = i, to = j, mode = “all”) for any i, j in gene list	*igraph* (1.3.5)
Correlation test	cor.test(i, j, alternative = "two.sided", method = “spearman”, continuity = T) for any i, j in gene list	R *stats* (4.2.3)
Gene sets and pathway resources	Cypher queries (Supp. Information)	docker.io/reactome/graphdb (version 90)

## Results

### Transcriptomic profiling of tissue samples from skeletal muscle biopsies from patients with irMyositis versus patients with Mi2 and TIF1γ subtype

Table 3 summarizes the characteristics of 35 patient samples used in this study. The cohort consists of 4 NDC samples, 8 Mi2 subtype samples, 6 TIF1γ subtype samples, and 17 irMyositis samples. We accounted for the technical variability effect of different NanoString Cartridges, sample conservation, and correlation between biopsies from the same patient using the *limma-voom* framework. We assessed the quality of batch-correction via principal component analysis (Supp. Information).

**Table 3:** Characteristics of muscle samples from the patient cohort used in this study (n = 35). The table displays the differences between the muscle samples that were considered in the analysis to reduce the effect of technical variability.

Sample	Group	Cartridge	Field-of-view (FOV)	Conservation method
1	NDC	C12	555	FFPE
2	NDC	C12	555	FFPE
3	NDC	C12	555	Cryopreserved
4	NDC	C12	555	Cryopreserved
5	Mi2 subtype	C49	194	Cryopreserved
6	Mi2 subtype	C49	194	Cryopreserved
7	Mi2 subtype	C15	555	Cryopreserved
8	Mi2 subtype	C15	555	Cryopreserved
9	Mi2 subtype	C15	555	Cryopreserved
10	Mi2 subtype	C15	555	Cryopreserved
11	Mi2 subtype	C15	555	Cryopreserved
12	Mi2 subtype	C15	555	Cryopreserved
13	TIF1γ subtype	C15	555	Cryopreserved
14	TIF1γ subtype	C15	555	Cryopreserved
15	TIF1γ subtype	C15	555	Cryopreserved
16	TIF1γ subtype	C15	555	Cryopreserved
17	TIF1γ subtype	C19	555	Cryopreserved
18	TIF1γ subtype	C20	555	Cryopreserved
19.1	irMyositis	C15	555	Cryopreserved
19.2	irMyositis	C49	194	Cryopreserved
20	irMyositis	C12	555	FFPE
21	irMyositis	C12	555	FFPE
22	irMyositis	C12	555	FFPE
23.1	irMyositis	C14	555	FFPE
23.2	irMyositis	C12	555	FFPE
24	irMyositis	C20	555	Cryopreserved
25	irMyositis	C20	555	Cryopreserved
26	irMyositis	C20	555	Cryopreserved
27	irMyositis	C20	555	Cryopreserved
28	irMyositis	C20	555	Cryopreserved
29	irMyositis	C20	555	Cryopreserved
30	irMyositis	C19	555	Cryopreserved
31	irMyositis	C48	555	Cryopreserved
32	irMyositis	C48	555	Cryopreserved
33	irMyositis	C49	194	Cryopreserved

We checked the expression quality of both *endogenous* and *housekeeping* genes probed by the “Cancer-Immune” NanoString panel. We excluded 135 *endogenous* genes from downstream DGE analysis, as their expression was above the average negative control signal in more than four samples. Next, we performed the analysis of variance in expression profiles of the remainder 595 genes across the 4 diagnosis groups (NDC, TIF1γ subtype, Mi2 subtype, and irMyositis). 93 genes showed significant F-statistics after 5% FDR p-value correction. Finally, we performed DGE analysis for each pair-wise comparison. Table 4 illustrates the per-comparison count of upregulated and downregulated genes after 5% FDR p-value correction. We found no DEG when comparing the TIF1γ subtype and Mi2 subtype.

**Table 4:** Number of DEGs per comparison.

Comparison	Number of upregulated genes	Number of downregulated genes
Mi2 subtype—NDC	24	30
TIF1γ subtype—NDC	27	28
irMyositis—NDC	17	18
TIF1γ subtype – Mi2	0	0
irMyositis – Mi2 subtype	19	24
irMyositis – TIF1γ subtype	11	23

### GSEA of reactome pathways highlights differences in active immunological pathways

**Fig. 2:**
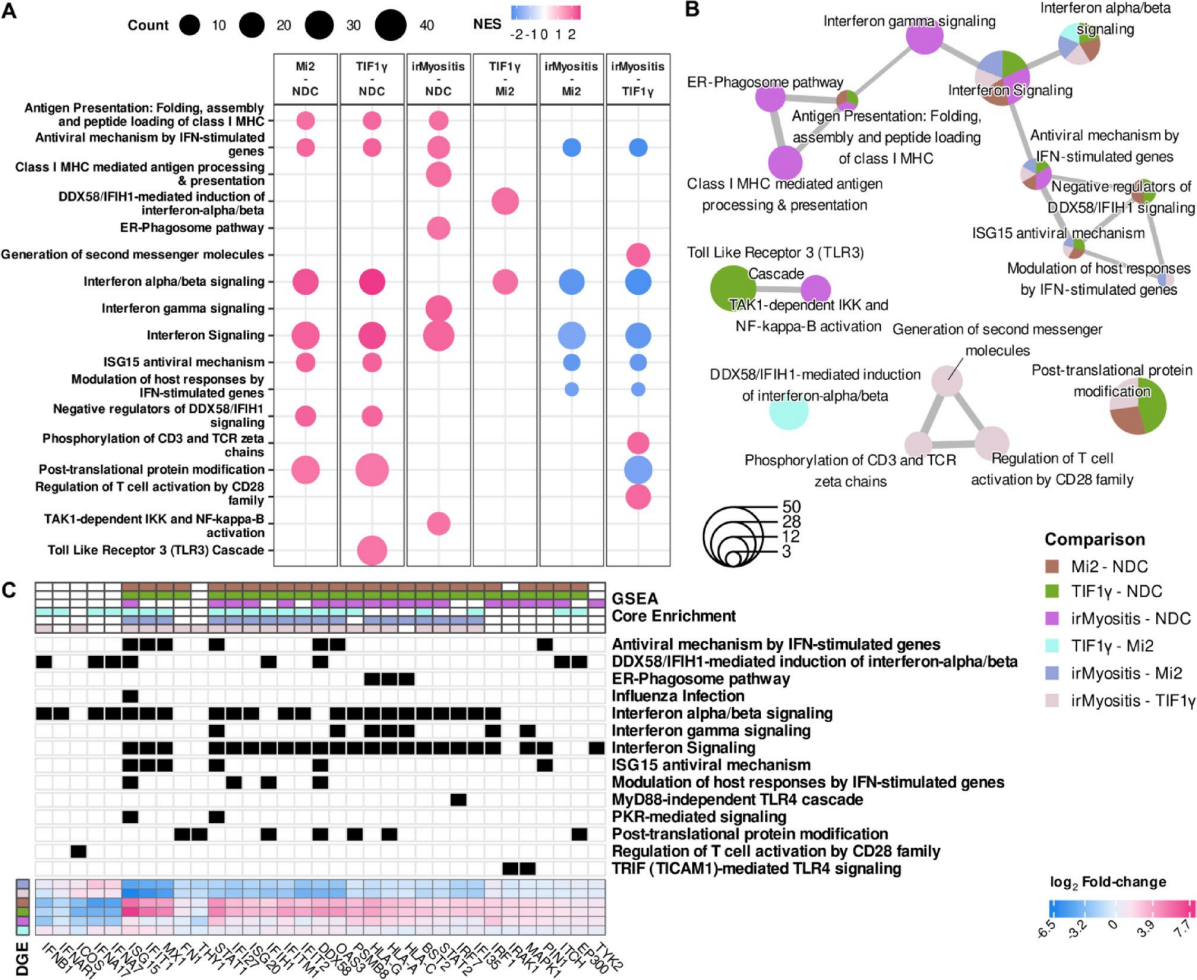
GSEA across irMyositis, Mi2 subtype, TIF1γ subtype, and NDC. **A**: Dot plot of significant enrichment scores for Reactome pathways, categorized by comparison (Benjamini–Hochberg correction – BH—5% FDR). Dot size refers to the number of genes belonging to the enrichment core, i.e., genes whose coordinated fold-change led to the significant gene set enrichment score (Count). Color depicts the GSEA enrichment score normalized by its expected value under the null hypothesis, given the pathway size (NES). **B**: Enrichment map of the significantly enriched pathways. Node sizes reflect the cardinalities of the union of unique genes belonging to enrichment cores of a pathway, while node sectors and their colors represent relative proportions of the respective enrichment core sizes in each comparison (GSEA). If the Jaccard similarity between unions of core enrichments for two distinct gene sets is higher than 0.2, an edge is drawn to represent a functional overlap between gene sets. **C**: Heatmap of 33 genes, selected based on belonging to at least one comparison-wise enrichment core (top blocks), and on showing a significant F-statistics at the same time (BH, 5% FDR). Correspondence between genes and enriched pathways is encoded by black rectangles. The genes are depicted and clustered column- and row-wise according to DGE (observed log2 fold-change). Discrete color codes for **B** and **C,** are annotated collectively (Comparison). To reduce the figure complexity, we have only depicted Reactome pathways listed within the top-nine absolute-value NES per comparison (**A-B**), and detailed annotation for the minimum set of enriched pathways that would include all selected genes (**C**).

Using the observed log2 fold-changes derived from the pairwise DGE analysis, we conducted GSEA for each pairwise comparison on Reactome pathways (Fig. 2). We observed opposite enrichment or Reactome pathways related to interferon signaling between DM and irMyositis (Fig. 2A). In particular, when compared to NDC, both Mi2 and TIF1γ subtypes showed positive enrichment of type-I IFN signaling as well as immune response by interferon-related genes classically described in the context of antiviral responses. However, these same pathways were negatively enriched in irMyositis when compared to DM. In contrast, type-II IFN signaling was positively enriched only in irMyositis compared to NDC. Interestingly, it appeared that the enrichments of *interferon alpha/beta signaling* and *DDX58/IFIH1-mediated induction of interferon alpha/beta* Reactome pathways were more pronounced in the TIF1γ subtype biopsies, compared to Mi2 subtype ones. Further comparison-specific, significant GSEAs were the positive enrichments of Toll-like receptor signaling in TIF1γ subtype compared to NDC, and T-cell specific regulations (CD28, CD3, and TCR molecular interactions) in irMyositis compared to TIF1γ subtype. The Jaccard similarity between core enrichment genes indicated that a subset of genes drove the enrichment of multiple, interrelated pathways (Fig. 2B). The heatmap in Fig. 2C clusters comparisons and enrichment core genes based on the fold-changes of the latter, whose coordinated fold-change led to a significant gene set enrichment score; the 33 genes shown, each belonging to one or multiple core enrichment groups, were selected due to their significant F-statistics. In particular, the genes *ISG15*, *STAT1*, *OAS3*, *IFI27*, *ISG20*, *IFITM1*, *DDX58*, and *BST2* contributed to gene set enrichment in all six comparisons and possessed qualitatively similar fold-change patterns. Reactome pathways related to interferon signaling are those with the highest coverage of genes with significant F-statistics.

### Pairwise differential gene expression reveals distinct regulatory patterns in irMyositis and Mi2 and TIF1γ subtypes

**Fig. 3:**
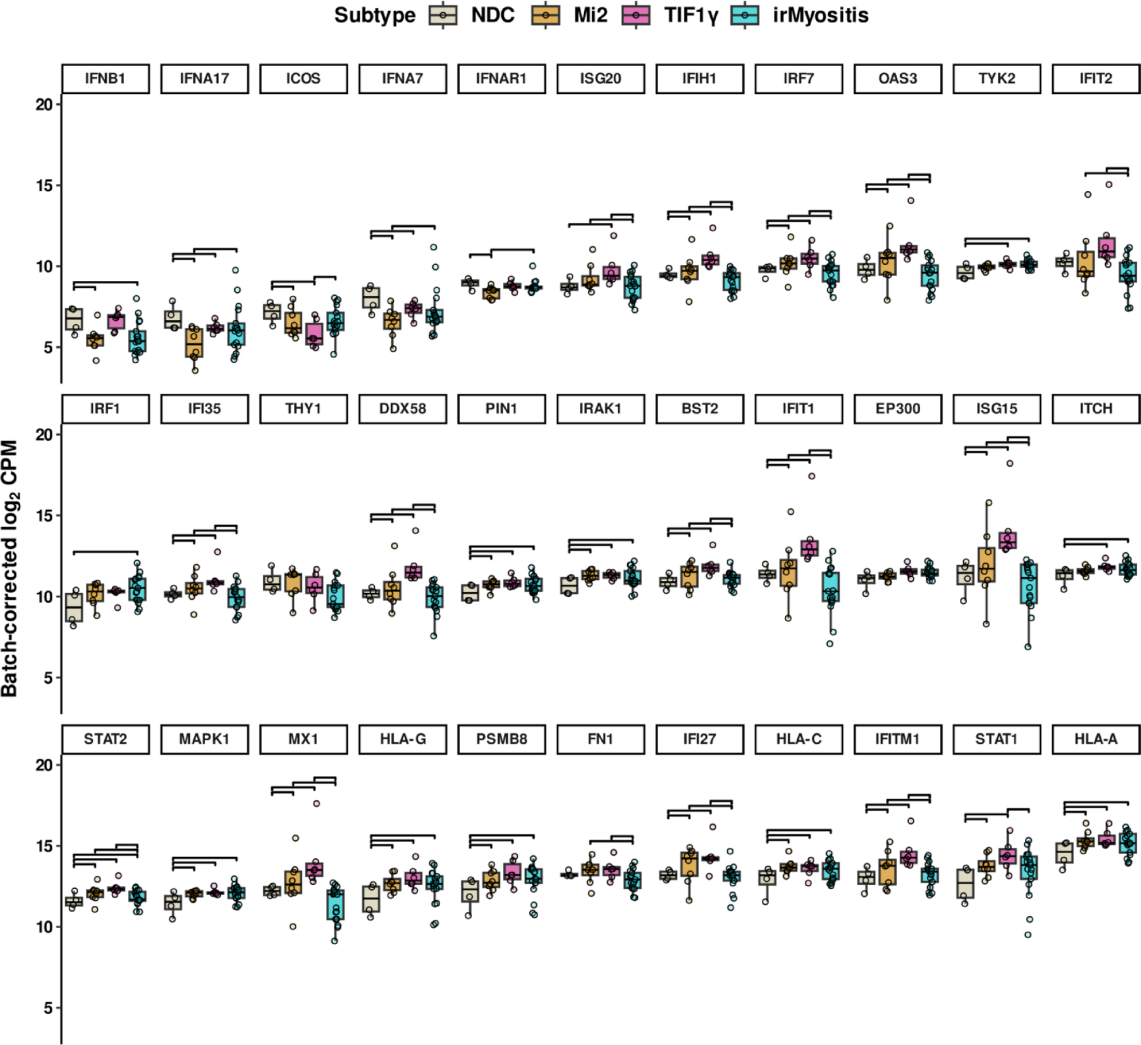
Pairwise differential expression of GSEA-driving genes. 33 genes were selected based on belonging to at least one comparison-wise enrichment core and on showing significant F-statistics at the same time (Benjamini–Hochberg correction, 5% FDR). Each box-and-whisker representation summarizes the batch-corrected, logarithmic counts-per-million (log_2_ CPM) of one gene in different sample groups (Subtype). Individual data points are also shown. The genes are sorted row-wise by average expression. IrMyositis, Mi2 subtype, TIF1γ subtype, and NDC are denoted by different colors. Horizontal brackets indicate significant pairwise DGE, after applying Benjamini–Hochberg correction (5% FDR, *hierarchical *post hoc correction – see Methods for further details).

For each gene that simultaneously drove the enrichment of multiple Reactome pathways and demonstrated statistically significant F-statistics (Fig. 2C), we investigated which pairwise comparisons between subtypes were also significant. This way, we identified groups of genes with distinct DGE patterns (Fig. 3). The RNA expression of the kinase-encoding genes *MAPK1* and *IRAK1*, the phosphorylation-dependent, peptidyl-prolyl cis/trans isomerase *PIN1,* the 20S immunoproteasome subunit *PSMB8*, and the class I MHC genes *HLA-A*, *HLA-C*, and *HLA-G* were upregulated in both DM and irMyositis compared to NDC. In contrast, many interferon-stimulated genes (ISGs) were upregulated in DM compared to NDC, but downregulated in irMyositis when compared to both Mi2 and TIF1γ subtypes; these differentially regulated ISGs encode for transcriptional regulators (*IRF7*), signal transduction and activation factors (*STAT1, ISG15, IFI27*, and *IFI35*), and effector and sensing proteins (*MX1*, *IFIT1*, *IFITM1*, *BST2*, *IFIH1*, *OAS3*, and *DDX58*). Interestingly, the extracellular-matrix component *FN1* was downregulated in irMyositis compared to both Mi2 and TIF1γ subtypes, but not upregulated in the latter two when compared to NDC.

**Fig. 4:**
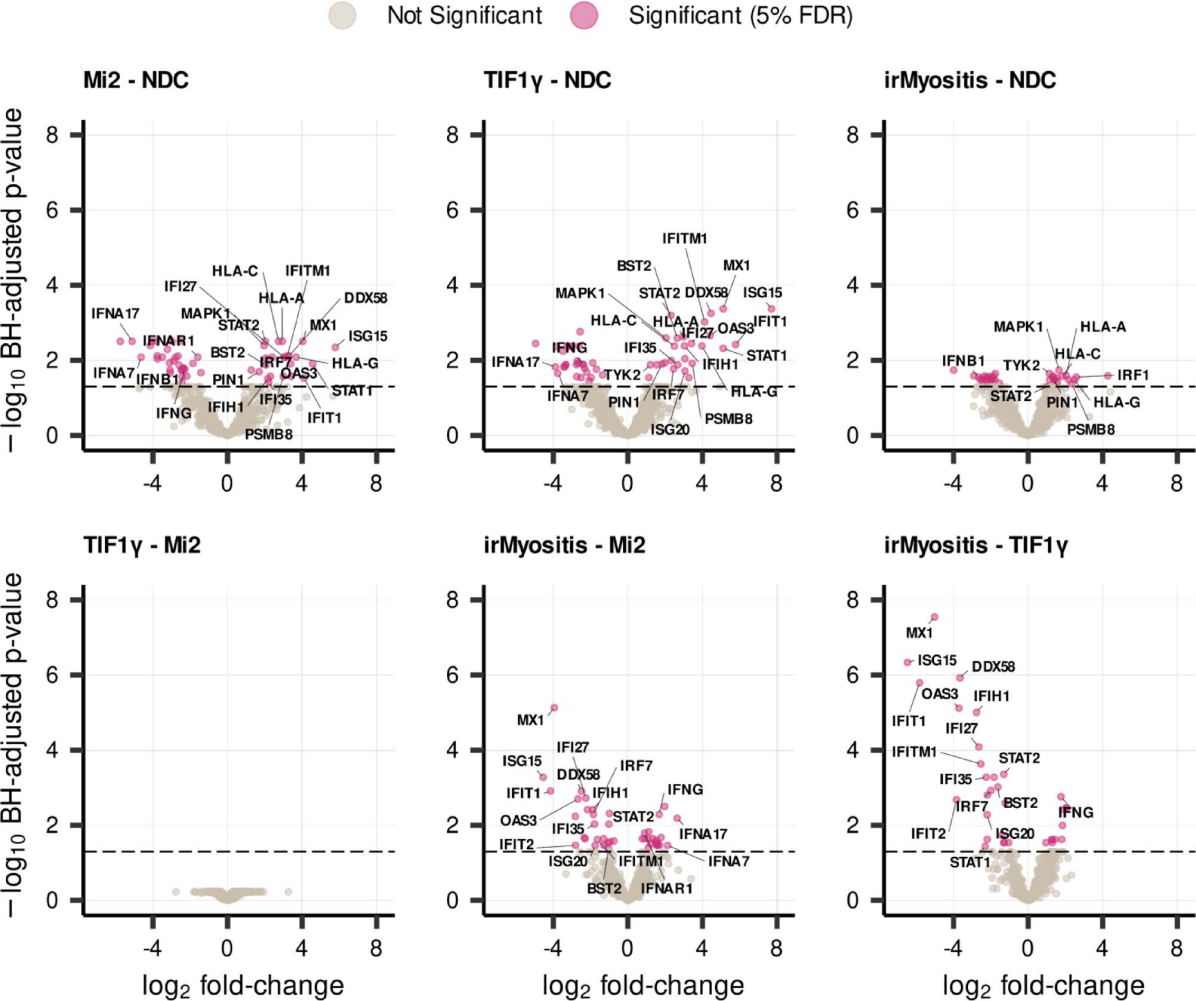
Differential expression patterns of genes related to interferon signaling. The volcano plots show Benjamini-Hochberg (BH) adjusted p-values and logarithmic fold-changes of 595 analyzed genes for each comparison. The horizontal dashed line corresponds to a log-transformed adjusted p-value of 0.05 and all genes above the line are significantly differentially expressed (5% FDR, *hierarchical *post hoc correction – see Methods for further details). Names of significant genes belonging to the Reactome pathways i) *Interferon signaling*, ii) *Interferon alpha/beta signaling*, iii) *Antiviral mechanisms by IFN-stimulated genes*, iv) *ISG15 antiviral mechanism*, and v) *Negative regulators of DDX58/IFIH1 signaling*. See Supp. Information for tabulated gene-to-pathway correspondence.

GSEA and pairwise differential expression of core enrichment genes highlighted interferon signaling and ISGs as the most prominent feature distinguishing DM and irMyositis (Fig. 2–3). To further inspect this outcome, we selected genes annotated under the GSEA-derived Reactome pathways i) *Interferon signaling*, ii) *Interferon alpha/beta signaling*, iii) *Antiviral mechanisms by IFN-stimulated genes*, iv) *ISG15 antiviral mechanism* and v) *Negative regulators of DDX58/IFIH1 signaling*. Then, we utilized the results of the post hoc test statistics for each of the six pairwise comparisons to identify all DEGs in the panel that also belong to these pathways, irrespective of GSEA core enrichments (Fig. 4 and Supp. Information). In addition to the previously described ISGs, we observed a consistent downregulation of type-II IFN in both TIF1γ and Mi2 subtypes compared to NDC, as well as its upregulation in irMyositis compared to DM. The volcano plots highlight that fewer ISGs were found to be differentially regulated in irMyositis compared to DM; nevertheless, irMyositis still shows upregulation of a handful of molecular components of interferon signaling such as *TYK2* and *IRF1*, when compared to NDC.

Interestingly, we found fold-change directions opposite to what GSEA suggested for a few interferon genes such as *IFNB1*, *IFNAR1*, and especially *INFA17* and *IFNA7*. However, it must be noted that their average expression was relatively low across the sampled biopsies and that the irMyositis group presented outliers in terms of log_2_ CPM of these genes (Fig. 3).

### Clustering of DEGs is conserved in both interaction network and expression correlation analyses

Next, we were interested in assessing if genes with similar DGE patterns were also closely associated in terms of mechanistic interactions or expression correlation with known cell type markers. In particular, we focused on genes that after post hoc testing showed i) significant regulation in the same direction in the two comparisons of irMyositis against Mi2 and TIF1γ subtypes, and ii) opposite regulation or insignificant DGE, in the two comparisons of Mi2 and TIF1γ subtypes against NDC. We defined such genes as putative immunotherapy-related DEGs (irDEGs). Table 5 lists irDEGs and their observed, significant fold-changes.

**Table 5:** Immunotherapy-related DEGs (irDEGs). Selection of irDEGs was based on i) significant regulation in the same direction in the two comparisons of irMyositis against Mi2 and TIF1γ subtypes, and ii) opposite regulation or insignificant DGE, in the two comparisons of Mi2 and TIF1γ subtypes against NDC, after post hoc testing. Observed expression was corrected for batch and conservation covariates and averaged across all samples. Observed fold-changes different from zero are indicated if significant after post hoc multiple testing adjustment of pairwise comparisons (5% FDR) – see Methods for further details.

Gene name	Average expression (log2 CPM)	Mi2—NDC	TIF1γ—NDC	irMyositis—NDC	TIF1γ—Mi2	irMyositis—Mi2	irMyositis—TIF1γ
BST2	11.50	2.10	2.60	0.00	0.00	− 1.04	− 1.62
CASP10	7.20	− 1.90	-1.70	0.00	0.00	1.12	0.95
CCL14	10.40	0.00	0.00	0.00	0.00	1.56	2.05
CFB	9.90	0.00	0.00	0.00	0.00	− 2.17	− 2.19
CR1	9.50	0.00	0.00	0.00	0.00	1.56	2.07
DDX58	10.50	3.30	4.50	0.00	0.00	− 2.49	− 3.66
FCGR1A	6.80	0.00	0.00	0.00	0.00	1.21	1.28
FN1	13.30	0.00	0.00	0.00	0.00	− 1.29	− 1.30
IFI16	11.80	0.00	0.00	0.00	0.00	− 1.07	1.05
IFI27	13.80	3.00	3.40	0.00	0.00	− 2.26	− 2.65
IFI35	10.20	2.20	2.70	0.00	0.00	− 1.79	− 2.25
IFIH1	9.80	2.20	3.00	0.00	0.00	− 1.90	− 2.77
IFIT1	11.80	4.10	5.80	0.00	0.00	− 4.15	− 5.82
IFIT2	10.20	0.00	0.00	0.00	0.00	− 2.81	− 3.84
IFITM1	13.90	3.20	4.10	0.00	0.00	− 1.63	− 2.54
IFNG	7.20	− 2.30	− 2.40	0.00	0.00	1.68	1.76
IL1RAP	8.90	0.00	0.00	0.00	0.00	− 0.97	− 1.24
IRF7	10.10	2.30	2.50	0.00	0.00	− 1.85	− 2.00
ISG15	11.90	5.80	7.70	0.00	0.00	− 4.55	− 6.47
ISG20	9.10	0.00	3.10	0.00	0.00	− 1.76	− 2.20
KLRC2	6.90	− 2.40	− 2.50	0.00	0.00	1.78	1.84
MX1	12.70	4.00	5.10	0.00	0.00	− 3.94	− 5.02
NT5E	10.40	0.00	0.00	-1.60	0.00	− 1.30	− 1.83
OAS3	10.40	3.40	4.40	0.00	0.00	− 2.68	− 3.71
STAT2	12.00	2.00	2.30	1.00	0.00	− 1.01	− 1.30
TGFB2	10.30	3.40	3.30	0.00	0.00	− 2.34	− 2.20
TIGIT	7.70	− 2.40	− 2.80	0.00	0.00	1.54	1.86
TNFRSF8	7.20	− 2.60	− 2.10	0.00	0.00	1.97	1.44

**Fig. 5:**
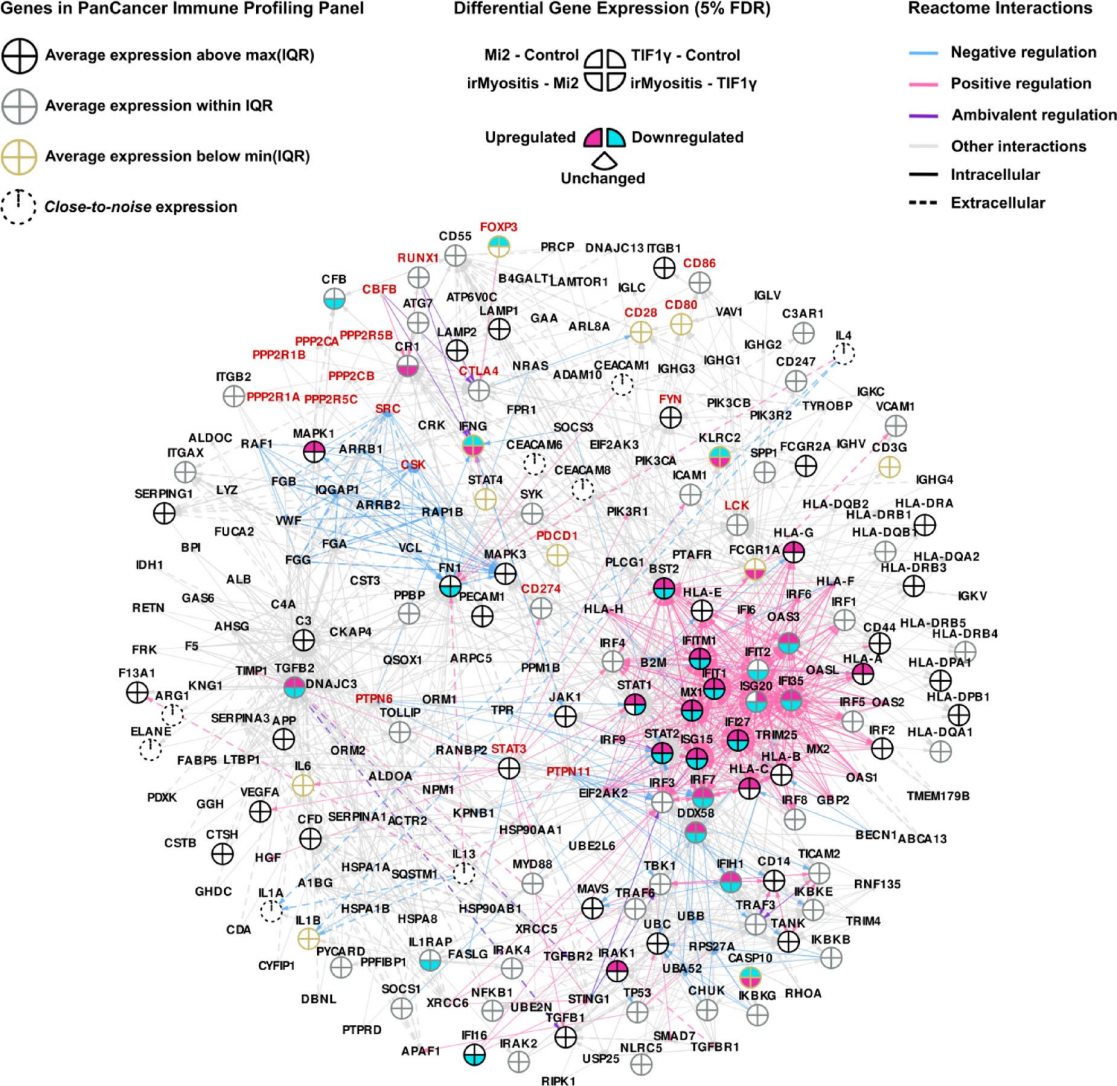
Interaction network of immunotherapy-related genes. Genes that after post hoc testing showed i) significant regulation in the same direction in the two comparisons of irMyositis against Mi2 and TIF1γ subtypes, and ii) opposite regulation or insignificant DGE, in the two comparisons of Mi2 and TIF1γ subtypes against NDC were selected and defined as irDEGs. Next, directional interactions from Reactome pathways containing irDEGs, (PD-L1), *PDCD1* (PD-1), or *CTLA4* were extracted. The depicted directed network consists of all-pairs shortest paths (APSPs) between such genes within the selected pathways. Genes included in the nCounter PanCancer Immune Profiling Panel are depicted by pie charts, while genes excluded from the analysis due to close-to-noise expression levels are depicted as dashed circles (see Methods for details). Each sector of the pie chart represents a pairwise DGE analysis between NDC, TIF1γ, Mi2, and irMyositis subtypes. Stroke color of pie charts represents the average gene expression across subtypes with respect to the pan-gene interquartile expression (IQR). Nodes of genes not included in the panel are minimized. *CD274*, *PDCD1*, *CTLA4*, and their neighbors are highlighted by red labels. Reactome annotation for the interaction types are depicted by the color and type of edge lines and the network is laid out using a force-directed algorithm in which stronger and weaker forces reflect intracellular and extracellular interactions (see Methods for details).

We also included in the interaction analysis the ICI targets CD274 (PD-L1), PDCD1 (PD-1), and CTLA4, whose DGE was statistically insignificant in all six comparisons (Supp. Information). Figure 5 illustrates the mechanistic interactions obtained after selecting all-pairs shortest paths (APSPs) within irDEGs and ICI targets from the Reactome Graph Database^29^. Unfortunately, NT5E and TNFRSF8 possessed no path to any other irDEG or ICI target, while CCL14 and TIGIT had not been curated in any Reactome pathway at the time of the analysis (April 2025). We employed the Fruchterman-Reingold algorithm to obtain a force-directed layout where directed intracellular and extracellular interactions are respectively assigned weak and strong spring-like repulsions forces, highlighting a dense hub of intracellular interactions between ISGSs. While most ISG interactions appeared as positively regulating, DDX58, IFIH1, and TRIM25 exhibited a cycle of negative regulations belonging to the pathway DDX58/IFIH1-mediated induction of interferon-alpha/beta (R-HSA-168928). Annotated under the same pathway, CASP10, a component of the death-inducing signaling complex (DISC), can participate in RIG-I (DDX58)/MDA5 (IFIH1)-dependent immune response; the reason for the observed deregulation pattern opposite to DDX58 at the RNA level is unclear, as it could imply higher levels in irMyositis of either its inactive pro-form or its active, cleaved one. FCGR1A, the gene encoding for Fc-gamma receptor 1, shares interactions with interferon response factors (IRFs) and several other genes such as B2M as a result of DNA binding by IFNG-activated factor to their promoter regions (GAF, ReactionLikeEvent R-HSA-1031716). However, its discordant regulation and lower average expression compared to IRFs may imply that its upregulation happened in a separate cell subpopulation, such as macrophages and dendritic cells that classically express Fc-gamma receptors. KLRC2, TGFB2, FN1, CR1, CFB, and IFNG were laid out further away from ISGs. In particular, IFNG is neighbored by RUNX1, CBFB, and PTPN6, which in turn directly interact with CTLA4, CD274, and PDCD1, making it one of the irDEGs with the shortest connections to all ICI therapy targets. RUNX1 and CBFB encode for transcription factors that can respectively promote transcription of IFNG and CR1 and inhibit that of CTL4A in the absence of FOXP3, a hallmark transcription factor in regulatory T cells (R-HSA-8877330). FOXP3 was downregulated in all three comparisons between myositis conditions and NDC (Supp. Information). Type-II IFN signaling can be hampered by protein tyrosine phosphatases (PTPs) in cells bearing type-II IFN receptors (R-HSA-877300), as reflected by the negative regulation from PTPN6 to IFNG. In fact, both SHP-1 (PTPN6) and SHP-2 (PTPN11) show negative regulation interactions in the APSP-derived network; binding of PD-1 (CD274) or CTLA4 to their respective ligands, causing phosphorylation in the former, can recruit and activate these PTPs in T cells (R-HSA-389948, R-HSA-389948).

**Fig. 6:**
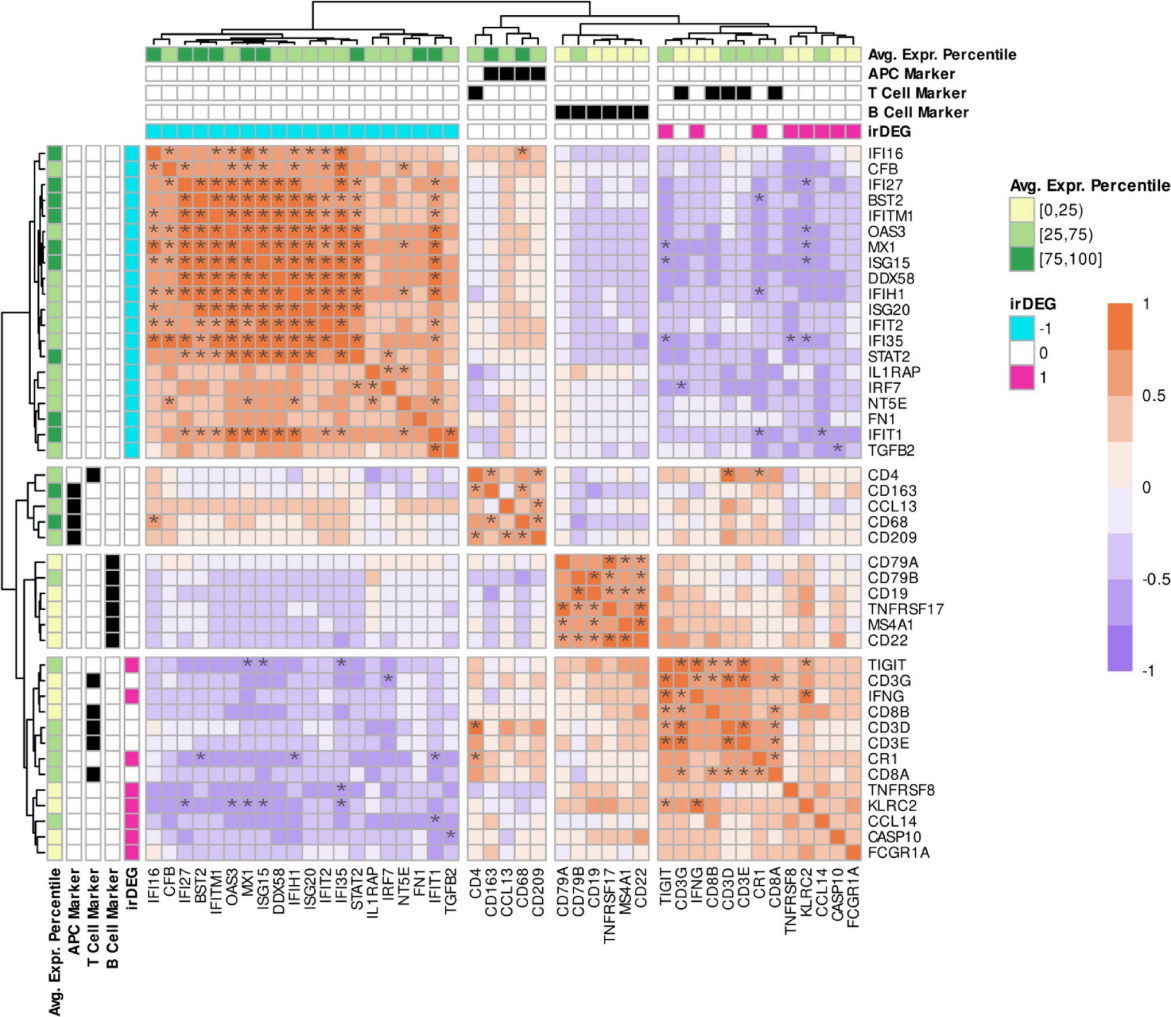
Correlation heatmap of immune cell biomarkers and immunotherapy-related genes. Genes that after post hoc testing showed i) significant regulation in the same direction in the two comparisons of irMyositis against Mi2 and TIF1γ subtypes, and ii) opposite regulation or insignificant DGE, in the two comparisons of Mi2 and TIF1γ subtypes against NDC were selected and defined as irDEGs. Turquoise rectangles correspond to irDEGs downregulated in irMyositis—DM comparisons, while red ones correspond to upregulated ones. T cell, B cell, and APC biomarkers were curated according to the whitepaper of the nCounter PanCancer Immune Profiling Panel and indicated by black rectangles. The green gradient represents the percentile range in which the average gene expression falls, with respect to the whole-panel expression. Correlation tests were performed using Spearman correlation and via asymptotic *t* distribution and Edgeworth series approximations. Significant correlation values (orange-purple gradient) are reported by asterisks (Benjamini–Hochberg correction, 5% FDR). An unsupervised cut of the hierarchical clustering with four as desired number of groups is applied.

Finally, we investigated expression correlation between irDEGs and immune cell gene markers provided by the nCounter PanCancer Immune Profiling Panel. The rationale was to assess if similarly expressed irDEGs clustered together with genes characteristic of a particular immune cell. Based on the 595 analyzed genes, we selected a) *GTF3C1*, *ZNF205*, *GZMB*, and *IL21R* as natural killer (NK) cell markers; b) *CD19*, *MS4A1*, *CD22*, *CD79A*, *CD79B*, and *TNFRSF17* as B cell markers; c) *CD3D*, *CD3E*, *CD3G*, *CD4*, *CD8A*, and *CD8B* as T cell markers; d) *CD68*, *CD163*, *CCL13*, and *CD209* as antigen-presenting cell (APC) markers. Figure 6 shows the heatmap we obtained by computing Spearman correlation between genes using batch-corrected log_2_ CPM values. In contrast to the other cell marker genes, The NK cell ones showed no significant, positive correlation with each other, and we therefore excluded them from the clustering analysis (Supp. Information). We applied an unsupervised cut of the hierarchically clustered dendrogram into four groups and observed clusters respectively consisting of i) irDEGs downregulated in irMyositis compared to DM and no immune cell marker; ii) APC markers and *CD4* iii) B cell markers; iv) irDEGs upregulated in irMyositis compared to DM and remaining T cell markers. The first cluster comprised most of the previously discussed ISGs, and we found that many of the observed Spearman positive correlations were also statistically significant (5% FDR). A smaller subset of downregulated irDEGs showed similar, but mostly insignificant correlations to ISG expression (*IL1RAP*, *IRF7*, *NT5N*, *FN1*, *TGFB2*). Cluster (iv) further bifurcated into one subset comprising T cell markers and the irDEGs *TIGIT*, *IFNG*, and *CR1*, the other consisting of the irDEGs *TNFRSF8*, *KLRC2*, *CCL14*, *CASP10*, and *FCGR1A*. Among the latter, *KLRC2* significantly correlated with *TIGIT* and *IFNG* expression, while the rest exhibited no significant, positive correlations.

## Discussion

ICI therapy is increasingly applied across a wide range of tumor entities and at earlier stages of tumor development. However, it can also cause irAE entailing morbidity and long-lasting symptoms that can persist after immunotherapy discontinuation and even be fatal. Thus, a better understanding of the pathological mechanisms underlying irAEs, such as irMyositis, is required. Although most patients with irMyositis respond well to corticosteroids, some can still develop sequelae and fatalities may occur^34^. In fact, irMyositis is one of the side effects with the highest mortality rate of around 20–46%^4,35^ and myalgic symptoms are among the most frequent chronic side effects even after cessation of ICI therapy^3^. Therefore, early diagnosis and initiation of treatment, as well as tailored therapeutic approaches for steroid-refractory cases, are essential. In this study, we characterized irMyositis compared to Mi2 and TIF1γ subtypes as well as NDC at a gene expression level using patient biopsies. Our study revealed diverse gene regulatory programs, thus giving insights into the immunological components that distinguish these diseases.

Based on our findings, irMyositis and Mi2/TIF1γ subtypes seem to show different molecular signatures related to interferon signaling. Using NanoString nCounter PanCancer Immune Profiling Panel, we were able to identify a consistent upregulation of ISGs related to type-I IFN signaling in DM compared to NDC and an opposite downregulation of the same ISGs in irMyositis compared to DM. Our analysis seemed to indicate that irMyositis does not possess the typical interferon signature that appears to characterize various DM and inflammatory idiopathic myopathies, including the MDA5 subtype which wasn’t included in our study^36,37^. Despite we observed a generalized overexpression of *PSMB8*, whose transcription has been associated to type-II IFN exposure, in all three comparisons between myositis and NDC conditions, *IFNG* was downregulated in DM compared to NDC and upregulated in irMyositis compared to DM. Interestingly, our network-based analysis recognized the interactions between *IFNG* and the effector PTPs activated by PD-1 and CTLA4 signaling as a potential hub through which the effects of ICI cancer treatment could lead to the observed differences between irMyositis and DM^38^. The fact that the master T cell regulator *FOXP3* was downregulated in all myositis conditions might imply, that cell subpopulations other than T cells could cause the characteristic regulation observed in irMyositis^39^. The hub of intracellularly interacting ISGs formed a consistent cluster of expression correlations that seem to suggest that mostly non-immune cells produce the regulated ISGs^40^. In contrast, *TIGIT* and *IFNG* clustered and positively correlated with T cell markers, but also with *KLRC2*, a C-type lectin receptor primarily expressed by NK cells^41^: an interplay between T and NK cells has been recently shown in inclusion body myositis^42^. A handful of irDEGs couldn’t be further characterized by our network- and correlation-based analysis. CD30 (*TNFRSF8*) has been shown to be downregulated in other autoimmune diseases: its upregulation would further set irMyositis apart from DM^43^. *NT5E* has been previously characterized as an immunosuppressive factor involved in response to ICI cancer therapy^44,45^. *TIGIT* (CD155) is a recently identified immune checkpoint expressed in both T and NK cell, in agreement with our expression correlation analysis: its higher expression in irMyositis compared to DM makes it a druggable candidate whose role in immunotherapy-induced conditions such as irMyositis should be further assessed in future studies^46^. Lastly, we observed *CCL14* upregulation exclusively in irMyositis-DM comparisons: this chemokine was previously studied in the context of cancer progression and associated with immune cell infiltration^47,48^.

We cross-compared the expression patterns of *FOXP3*, *IFNG*, and the other identified irDEGs using a publicly available RNAseq dataset (GSE220915) of different spontaneous myositis types and healthy muscle biopsies, and semi-automatic text mining and review of research article on myositis, ICI, and irAE using ENQUIRE (Methods and Supp. Information)^32,33^. The gene-wise average expression strongly correlates between our dataset and GSE220915. The observed irDEG fold-changes between Mi2/TIF1γ subtypes and NDC clustered the closest to those reported in GSE220915 between DM and healthy controls, in contrast to comparisons between irMyositis and the other subtypes in our study, confirming the difference between irMyositis and DM. However, we observed discrepancies in fold-change directions of lowly-expressed genes such as *FOXP3* and *IFNG*, and close-to-noise expression of IFN genes in GSE220915 that also possess high variability in our dataset, such as *IFN*A7, *IFNA17*, and *IFNB1*. We think these differences can be attributed to the choice of healthy control biopsies and to the exact protocol followed to select the slices, as differences in the latter can have a considerable impact on the outcome of cell-specific, generally lowly expressed genes such as interferons and *FOXP3*. Nevertheless, literature text mining highlighted the over-expression of type-II IFN and the localized enrichment of Th1 and CD8^+^ leukocytes over Th2 and FOXP3^+^ ones in the context of ICI therapy and irAE^49–51^. At the same time, type-I IFN levels in serum were found to correlate with medical assessment of DM severity and an RNAseq study including irMyositis and DM found enrichment of type-I IFN signaling in both but type-II signaling only in irMyositis^13,52^.

A limitation of this study is that a subgroup of patients received corticosteroids prior to undergoing muscle biopsy. This may have influenced gene expression results, i.e. by downregulating pro-inflammatory genes (TNF, IL1B, IL6, PTGS2, components of the iNOS complex, HLA genes, FBXO32, and TRIM63)^53^. However, it would have been unethical to delay patient treatment, in cases where muscle biopsies could not be scheduled promptly. Another limitation is the bulk processing of total RNA from biopsies, therefore losing both single-cell and spatial information of the gene expression. We tried to alleviate this shortcoming by combining a molecular interaction analysis with expression correlation tests using immune cell markers provided in the NanoString panel^31^. Also, for lowly expressed genes like *IFNA7* and *IFNB1* with high variability and borderline detection, bulk analyses can limit the interpretation of gene expression patterns. Nevertheless, the NanoString nCounter assay has been shown to be as sensitive as qPCR and their gene expression estimates strongly correlated in previous independent studies on interferonopathies, blood, and tumor samples, including FFPE biopsies, when performed on the same total RNA extract^54–56^.

To summarize, our results highlighted gene regulations (type-II IFN signaling) and putative effectors (T and NK cells) that can differentiate irMyositis and DM and be directly affected by ICI therapy, thus offering further avenues for understanding immunotherapy-induced autoimmune conditions. We think that future studies at cellular resolution, such as single-cell sequencing or spatial transcriptomics, will allow to better characterize the differences between myositis subtypes.

## Electronic supplementary material

Below is the link to the electronic supplementary material.

Supplementary Material 1

## Data Availability

Data is provided within the manuscript or in supplementary information files (Supplementary_material.zip). The compressed archive contains RCC files, a condensed sample information file, Reactome-derived gene sets and multigraphs used for GSEA and network-based analysis (Figs. 2 and 5), complete differential gene expression and gene set enrichment analysis results (.xlsx file), graph databases of textmined literature used for cross-comparison (tar.gz files), as well as code and additional results described in the manuscript (Supp. Information), in the form of an RMarkdown file exported in HTML format.
