# Supplementary material for: Gene expression and molecular pathway analyses differentiate immunotherapy-induced myositis from spontaneous dermatomyositis
Source: Sci Rep. 2025 Aug 4;15:28434. doi: 10.1038/s41598-025-11944-5 (PMC12322072; doi:10.1038/s41598-025-11944-5)
Supplement: Supplementary file 1 — Supplementary Material 1 [file 41598_2025_11944_MOESM1_ESM.zip › Supplementary_material/Data_Analysis_Code_and_Supplementary_Information.html]

Immunotherapy-induced myositis shows distinct immunological pathways from spontaneous MI-2+ and TIF-1γ+ dermatomyositis - Revision


Code 

- Show All Code
- Hide All Code

# Immunotherapy-induced myositis shows distinct immunological pathways from spontaneous MI-2+ and TIF-1γ+ dermatomyositis - Revision

#### Luca Musella

#### 23 June, 2025

- 1 R libraries to be imported
- 2 Data Preparation
  - 2.1 Import Sample Annotation and .RCC files
  - 2.2 Prepare Count Table
- 3 Statistical Analysis
  - 3.1 Differential Gene Expression
  - 3.2 Post-correction PCA
  - 3.3 Gene Set Enrichment Analysis (GSEA)
- 4 Inspect core enrichment of genes
  - 4.1 BoxPlots for each F-statistics-significant gene in a GSEA core-enrichment
- 5 Inspect DGE of genes related to Interferon signalling
- 6 All irDEGs and ICI targets APSPs
- 7 Expression Correlation between DEGs and Cell Markers
- 8 Comparison of irDGE results to other studies
  - 8.1 Using dataset GSE220915
  - 8.2 Using literature mining (ENQUIRE)
- 9 Save Data to Excel File

# 1 R libraries to be imported

The `sessionInfo()` output reported below provides information to replicate the working environment used in this study:

```
library(nanostringr)
library(basetheme)
library(stringr)
library(dplyr)
library(tidyverse)
library(ggpubr)
library(readxl)
library(tibble)
library(Biobase)
library(car)
library(stringr)
library(BiocManager)
library(parallel)
library(EnhancedVolcano)
library(clusterProfiler)
library(randomcoloR)
library(limma)
library(edgeR)
library(openxlsx)
library(qgraph)
library(igraph)
library(ggplot2)
library(patchwork)
library(ggfortify)
library(ComplexHeatmap)
library(GEOquery)
sessionInfo()
# R version 4.2.3 (2023-03-15)
# Platform: x86_64-pc-linux-gnu (64-bit)
# Running under: Ubuntu 24.04.2 LTS
# 
# Matrix products: default
# BLAS:   /home/vera/eberhamn/lib/R-4.2.3/lib/libRblas.so
# LAPACK: /home/vera/eberhamn/lib/R-4.2.3/lib/libRlapack.so
# 
# locale:
#  [1] LC_CTYPE=en_US.UTF-8       LC_NUMERIC=C               LC_TIME=en_US.UTF-8        LC_COLLATE=en_US.UTF-8    
#  [5] LC_MONETARY=en_US.UTF-8    LC_MESSAGES=en_US.UTF-8    LC_PAPER=en_US.UTF-8       LC_NAME=C                 
#  [9] LC_ADDRESS=C               LC_TELEPHONE=C             LC_MEASUREMENT=en_US.UTF-8 LC_IDENTIFICATION=C       
# 
# attached base packages:
# [1] grid      parallel  stats     graphics  grDevices utils     datasets  methods   base     
# 
# other attached packages:
#  [1] GEOquery_2.66.0        ComplexHeatmap_2.14.0  ggfortify_0.4.16       patchwork_1.3.0        igraph_1.3.5          
#  [6] qgraph_1.9.4           openxlsx_4.2.5.1       edgeR_3.40.2           limma_3.54.0           randomcoloR_1.1.0.1   
# [11] clusterProfiler_4.6.2  EnhancedVolcano_1.16.0 ggrepel_0.9.2          BiocManager_1.30.19    car_3.1-1             
# [16] carData_3.0-5          Biobase_2.58.0         BiocGenerics_0.44.0    readxl_1.4.3           ggpubr_0.5.0          
# [21] lubridate_1.9.3        forcats_1.0.0          purrr_1.0.2            readr_2.1.5            tidyr_1.3.1           
# [26] tibble_3.2.1           ggplot2_3.5.2          tidyverse_2.0.0        dplyr_1.1.4            stringr_1.5.0         
# [31] basetheme_0.1.3        nanostringr_0.1.0     
# 
# loaded via a namespace (and not attached):
#   [1] utf8_1.2.2             tidyselect_1.2.1       RSQLite_2.2.20         AnnotationDbi_1.60.0   htmlwidgets_1.6.1     
#   [6] BiocParallel_1.32.6    Rtsne_0.16             scatterpie_0.2.1       munsell_0.5.0          codetools_0.2-18      
#  [11] withr_2.5.0            colorspace_2.1-0       GOSemSim_2.24.0        knitr_1.41             rstudioapi_0.17.1     
#  [16] stats4_4.2.3           ggsignif_0.6.4         DOSE_3.24.2            GenomeInfoDbData_1.2.9 mnormt_2.1.1          
#  [21] polyclip_1.10-4        bit64_4.0.5            farver_2.1.1           downloader_0.4         vctrs_0.6.5           
#  [26] treeio_1.22.0          generics_0.1.3         gson_0.1.0             xfun_0.39              timechange_0.2.0      
#  [31] doParallel_1.0.17      R6_2.5.1               GenomeInfoDb_1.34.9    clue_0.3-64            graphlayouts_1.0.0    
#  [36] locfit_1.5-9.7         bitops_1.0-7           cachem_1.0.6           fgsea_1.24.0           gridGraphics_0.5-1    
#  [41] scales_1.3.0           ggraph_2.1.0           nnet_7.3-18            enrichplot_1.18.4      gtable_0.3.1          
#  [46] tidygraph_1.2.3        rlang_1.1.3            GlobalOptions_0.1.2    splines_4.2.3          rstatix_0.7.2         
#  [51] lazyeval_0.2.2         broom_1.0.5            checkmate_2.2.0        yaml_2.3.6             reshape2_1.4.4        
#  [56] abind_1.4-5            backports_1.4.1        qvalue_2.30.0          Hmisc_5.0-1            tools_4.2.3           
#  [61] bookdown_0.34          psych_2.3.3            lavaan_0.6-15          ggplotify_0.1.0        ellipsis_0.3.2        
#  [66] jquerylib_0.1.4        RColorBrewer_1.1-3     Rcpp_1.0.9             plyr_1.8.8             base64enc_0.1-3       
#  [71] zlibbioc_1.44.0        RCurl_1.98-1.9         rpart_4.1.19           GetoptLong_1.0.5       pbapply_1.7-0         
#  [76] viridis_0.6.2          cowplot_1.1.1          S4Vectors_0.36.1       cluster_2.1.8.1        magrittr_2.0.3        
#  [81] data.table_1.14.6      circlize_0.4.15        matrixStats_1.0.0      hms_1.1.2              evaluate_0.19         
#  [86] HDO.db_0.99.1          jpeg_0.1-10            shape_1.4.6            IRanges_2.32.0         gridExtra_2.3         
#  [91] compiler_4.2.3         V8_4.3.0               crayon_1.5.2           shadowtext_0.1.2       htmltools_0.5.4       
#  [96] ggfun_0.1.0            corpcor_1.6.10         tzdb_0.4.0             Formula_1.2-5          aplot_0.1.10          
# [101] DBI_1.1.3              tweenr_2.0.2           formatR_1.14           MASS_7.3-60.0.1        Matrix_1.5-3          
# [106] cli_3.6.2              quadprog_1.5-8         pkgconfig_2.0.3        foreign_0.8-84         xml2_1.3.3            
# [111] foreach_1.5.2          pbivnorm_0.6.0         ggtree_3.6.2           bslib_0.4.2            XVector_0.38.0        
# [116] yulab.utils_0.0.6      digest_0.6.31          Biostrings_2.66.0      rmarkdown_2.19         cellranger_1.1.0      
# [121] fastmatch_1.1-3        tidytree_0.4.2         htmlTable_2.4.1        curl_4.3.3             gtools_3.9.4          
# [126] rjson_0.2.21           lifecycle_1.0.3        nlme_3.1-161           glasso_1.11            jsonlite_1.8.4        
# [131] viridisLite_0.4.1      fansi_1.0.3            pillar_1.9.0           lattice_0.20-45        KEGGREST_1.38.0       
# [136] fastmap_1.1.0          httr_1.4.4             GO.db_3.16.0           glue_1.6.2             zip_2.2.2             
# [141] fdrtool_1.2.17         iterators_1.0.14       png_0.1-8              bit_4.0.5              ggforce_0.4.1         
# [146] stringi_1.8.7          sass_0.4.4             blob_1.2.3             memoise_2.0.1          ape_5.7-1
```

After installing `BiocManager` version 3.16, you can install all required libraries via BiocManager::install(c(‘GEOquery’,‘ComplexHeatmap’,‘ggfortify’,‘patchwork’,‘igraph’,‘qgraph’,‘openxlsx’,‘edgeR’,‘limma’,‘randomcoloR’,‘clusterProfiler’,‘EnhancedVolcano’,‘ggrepel’,‘BiocManager’,‘car’,‘carData’,‘Biobase’,‘BiocGenerics’,‘readxl’,‘ggpubr’,‘lubridate’,‘forcats’,‘purrr’,‘readr’,‘tidyr’,‘tibble’,‘ggplot2’,‘tidyverse’,‘dplyr’,‘stringr’,‘basetheme’,‘nanostringr’)).

These are the R functions used in this study, sorted by category:

```
pckgs = sessionInfo()$otherPkgs

basepckgs = sessionInfo()$basePkgs

funbypckg = NCmisc::list.functions.in.file("~/mount-srv1/musellla/code/R/musellla_R_workspace/Colitis_MelAutim/Myositis_Revision_backup.R")

names(funbypckg) = stringr::str_remove(names(funbypckg), "^package\\:")

funbypckg = funbypckg[intersect(names(funbypckg), c(basepckgs, names(pckgs)))]

pckgs = pckgs[names(funbypckg)]

pckgs = pckgs[sort(names(pckgs))]
funbypckg = funbypckg[sort(names(funbypckg))]

categories = c("Utilities", "Plotting", "Plotting", "Code Parallelization", "Miscellaneous Statistical Utilities, Clustering, and Matrix Manipulation",
    "Utilities, File Reading and Writing", "Gene Set Enrichment Analysis", "Plotting", "Utilities", "Count Data Processing and Normalization",
    "Plotting", "Plotting", "Plotting", "Network reconstruction, analysis, and representation", "Linear Model Fit, Batch Correnction, and Differential Gene Expression",
    "RCC file parsing", "Results Storing", "Plotting", "Plotting", "Plotting", "Plotting", "Utilities", "Utilities")

basepckgs = intersect(names(funbypckg), basepckgs)

funpckdf = data.frame(Package = c(basepckgs, sapply(pckgs, "[[", "Package")), Version = c(rep("R 4.2.3 base package", length(basepckgs)),
    sapply(pckgs, "[[", "Version")), Category = categories, Functions = sapply(funbypckg[match(c(basepckgs, sapply(pckgs,
    "[[", "Package")), names(funbypckg))], paste0, collapse = ", "))

funpckdf = funpckdf[order(funpckdf$Category), ]

DT::datatable(funpckdf, caption = "R functions used, sorted by package and category", rownames = F, escape = F, extensions = c("FixedColumns",
    "FixedHeader"), options = list(scrollX = F, paging = T, fixedHeader = T, lengthMenu = list(c(-1), c("All"))))
```

# 2 Data Preparation

## 2.1 Import Sample Annotation and .RCC files

In this section we import into R sample information from the CSV containing clinical as well as technical information, and RCC raw count files using the functions `nanostringr::parse_counts()` and `nanostringr::parse_attributes()`. Make sure to `setwd()` to the location where you put the `RCC` folder containing the count data.


```
setwd("~/mount-srv1/musellla/PhD_Projects/MelAutim-Colitis-FACS-NanoString/DATA/Myositis")
sampinf = read.csv("~/mount-srv1/musellla/PhD_Projects/MelAutim-Colitis-FACS-NanoString/DATA/Myositis/IRMyositis_Sample_information_UKER_REVISION.csv")

sampinf$Subtype = str_replace_all(sampinf$Subtype, "PD1", "irMyositis")

sampinf$Subtype = str_replace_all(sampinf$Subtype, "TIF1g", "TIF1γ")

sampinf$Subtype = str_replace_all(sampinf$Subtype, "Control", "NDC")

sampinf = sampinf[sampinf$Tissue == "skelm", ]


sdir = list.dirs(full.names = F, recursive = F)[grepl("^RCC", list.dirs(full.names = F, recursive = F))]
sampinf$FileNameFull = file.path("RCC", sampinf$FileName)


nandat = lapply(sampinf$FileNameFull, function(RCC) (list(counts = parse_counts(RCC), attributes = parse_attributes(RCC))))
names(nandat) = sampinf$Biomarker.Nr.
panels = lapply(nandat, "[[", "attributes")
paneldf = apply(as.matrix(names(panels[[1]])), 1, function(att) (sapply(panels, "[[", att)))
colnames(paneldf) = names(panels[[1]])
paneldf = as.data.frame(paneldf)
paneldf$Biomarker.Nr. = rownames(paneldf)
sampinf = merge(sampinf, paneldf, by = "Biomarker.Nr.")

sampinf$Subtype = factor(sampinf$Subtype, levels = c("NDC", "Mi2", "TIF1γ", "irMyositis"))
```

```
knitr::kable(cbind(data.frame(`Sample Number` = seq(nrow(sampinf)), check.names = F), sampinf[, c("Subtype", "Cartridge",
    "detailed.origin", "Conservation")]), format = "pandoc", digits = 2, align = "l", caption = "Sample Information.", label = NA)
```

Sample Information.

| Sample Number | Subtype | Cartridge | detailed.origin | Conservation |
| --- | --- | --- | --- | --- |
| 1 | irMyositis | C12 | Zürich | FFPE |
| 2 | irMyositis | C12 | Essen-3 | FFPE |
| 3 | irMyositis | C12 | Essen-2 | FFPE |
| 4 | NDC | C12 | Erlangen - Pathologie | FFPE |
| 5 | NDC | C12 | Erlangen - Pathologie | FFPE |
| 6 | irMyositis | C12 | Darmstadt | FFPE |
| 7 | NDC | C12 | Berlin | Cryo |
| 8 | NDC | C12 | Berlin | Cryo |
| 9 | irMyositis | C14 | Erlangen - Dermatologie | FFPE |
| 10 | Mi2 | C15 | Berlin | Cryo |
| 11 | Mi2 | C15 | Berlin | Cryo |
| 12 | Mi2 | C15 | Berlin | Cryo |
| 13 | Mi2 | C15 | Berlin | Cryo |
| 14 | Mi2 | C15 | Berlin | Cryo |
| 15 | Mi2 | C15 | Berlin | Cryo |
| 16 | TIF1γ | C15 | Berlin | Cryo |
| 17 | TIF1γ | C15 | Berlin | Cryo |
| 18 | TIF1γ | C15 | Berlin | Cryo |
| 19 | TIF1γ | C15 | Berlin | Cryo |
| 20 | TIF1γ | C19 | Berlin | Cryo |
| 21 | TIF1γ | C20 | Berlin | Cryo |
| 22 | irMyositis | C15 | Berlin | Cryo |
| 23 | irMyositis | C20 | Berlin | Cryo |
| 24 | irMyositis | C20 | Berlin | Cryo |
| 25 | irMyositis | C20 | Berlin | Cryo |
| 26 | irMyositis | C20 | Berlin | Cryo |
| 27 | irMyositis | C20 | Berlin | Cryo |
| 28 | irMyositis | C20 | Berlin | Cryo |
| 29 | irMyositis | C19 | Berlin | Cryo |
| 30 | irMyositis | C48 | Erlangen - Neuropathologie | Cryo |
| 31 | irMyositis | C48 | Göttingen | Cryo |
| 32 | Mi2 | C49 | Berlin | FFPE |
| 33 | Mi2 | C49 | Berlin | FFPE |
| 34 | irMyositis | C49 | Berlin | FFPE |
| 35 | irMyositis | C49 | Berlin | FFPE |

```
ggplot(data = data.frame(table(sampinf$Subtype), x = rep("Subtype", length(table(sampinf$Subtype))))) + geom_bar(position = "stack",
    stat = "identity", aes(fill = Var1, y = Freq, x = x)) + theme_minimal() + scale_shape_manual(values = c(16, 15)) + theme(legend.title = element_text(color = "black",
    face = "bold"), title = element_text(color = "black", face = "bold"), strip.text = element_text(size = 8), axis.text.x = element_text(color = "black",
    face = "bold"), axis.title = element_blank(), legend.text = element_text(size = 12)) + scale_fill_manual(values = c("wheat3",
    "orange3", "maroon3", "turquoise3")) + scale_y_continuous(breaks = unname(cumsum(rev(table(sampinf$Subtype)))))
```

## 2.2 Prepare Count Table

In this section we aggregate sample-wise count data to construct the count matrix. Together with NanoString’s gene probe attributes and sample information, we then construct a `DGEList` object. We normalize for library size, then we apply CodeSet content normalization, i.e.

\1. Calculate the geometric mean of the selected housekeeping genes for each lane.
\2. Calculate the arithmetic mean of these geometric means for all sample lanes.
\3. Divide this arithmetic mean by the geometric mean of each lane to generate a lane-specific
normalization factor.
\4. Multiply the counts for every gene by its lane-specific normalization factor.

```
colexp = lapply(lapply(nandat, "[[", "counts"), function(df) (setNames(df[, c(4), drop = T], df$Name)))
colexp = lapply(colexp, function(v) (data.frame(gene = names(v), count = v)))


colcount = colexp %>%
    purrr::reduce(full_join, by = "gene")
colcount = column_to_rownames(colcount, "gene")
colnames(colcount) = names(colexp)
customprobes = colcount[!(complete.cases(colcount)), ]
colcount = colcount[(complete.cases(colcount)), ]
addinfo = nandat$`001 Muskel`$counts[match(rownames(colcount), nandat$`001 Muskel`$counts$Name), c("Code.Class", "Name",
    "Accession")]
colcount = cbind(addinfo, colcount)


y <- DGEList(counts = colcount[, match(make.names(sampinf$Biomarker.Nr.), make.names(colnames(colcount)), nomatch = NULL)],
    group = sampinf$Subtype, samples = tibble::column_to_rownames(sampinf, var = "Biomarker.Nr."), genes = colcount[, 1:3],
    lib.size = apply(colcount[, match(make.names(sampinf$Biomarker.Nr.), make.names(colnames(colcount)), nomatch = NULL)],
        2, sum))

y = calcNormFactors(y, method = "none")

negxsamp = cpm(y, normalized.lib.sizes = T, log = T, prior.count = 1)

negxsamp = negxsamp[grepl("NEG", rownames(negxsamp)), ]

print(paste("Median log2 CPM of negative controls:", signif(median(negxsamp), 3)))
```

```
# [1] "Median log2 CPM of negative controls: 4.35"
```

```
nccpms = apply(negxsamp, 2, mean)

print(paste("Average negative control log2 CPM across samples:", signif(mean(nccpms), 3), "±", signif(sd(nccpms), 3)))
```

```
# [1] "Average negative control log2 CPM across samples: 4.44 ± 0.735"
```

```
fbe = edgeR::filterByExpr(y, group = y$samples$group, min.count = 2^(mean(apply(negxsamp, 2, mean)) + 2 * sd(apply(negxsamp,
    2, mean))))


bad = rownames(y[!(fbe), ])


y = y[fbe & !(grepl("Positive|Negative", y$genes$Code.Class)), , keep.lib.sizes = F]

print(c("Number of 'endogenous' and 'housekeeping' genes retained after negative-control-based filtering", table(y$genes$Code.Class)))
```

```
#                                                                                                   
# "Number of 'endogenous' and 'housekeeping' genes retained after negative-control-based filtering" 
#                                                                                        Endogenous 
#                                                                                             "595" 
#                                                                                      Housekeeping 
#                                                                                              "40"
```

```
y = calcNormFactors(y, method = "none")


gmhkfactors = apply(y$counts, 2, function(v) (exp(mean(log(v[grepl("Housekeeping", y$genes$Code.Class)] + 1)))))


gmmhk = mean(gmhkfactors)


hkfactors = apply(y$counts, 2, function(v) (gmmhk/(exp(mean(log(v + 1))))))


y = y[!grepl("Housekeeping", y$genes$Code.Class), , keep.lib.sizes = F]

y$counts = t(apply(y$counts, 1, function(v) (v * hkfactors)))


hkcount = cpm(y, log = T, normalized.lib.sizes = T, prior.count = 1)

print(paste("How many genes were removed?", length(bad[!grepl("NEG|POS", bad)])))
```

```
# [1] "How many genes were removed? 135"
```

```
print(paste("IFN* genes removed because below background noise:", paste0(bad[grepl("IFN", bad)], collapse = " ")))
```

```
# [1] "IFN* genes removed because below background noise: IFNA2 IFNA8 IFNL2 IFNA1"
```

```
colcountraw = colcount
okgenes = setdiff(rownames(hkcount), bad)
hkcount = hkcount[match(okgenes, rownames(hkcount)), ]
plot(apply(hkcount, 1, sd) ~ apply(hkcount, 1, mean), xlab = "Avg. CPM post CodeSet Content normalization", ylab = "SD of CPM post CodeSet Content normalization")
```

# 3 Statistical Analysis

## 3.1 Differential Gene Expression

```
modm = sampinf
rownames(modm) = sampinf$`Master-ID`
colnames(modm) = str_replace(colnames(modm), " |[^[:alnum:]]", replacement = "")
counts = hkcount
tag = "irC_vs_UC_test"
rowdat = addinfo[match(rownames(counts), addinfo$Name), ]
rowdat = column_to_rownames(rowdat, "Name")
vars = c("Subtype", "cartridgeID", "fovcount", "Conservation", "CENTERBIOPSY")
wb <- createWorkbook()
rownames(modm) = modm$BiomarkerNr.

group = factor(y$samples$group, levels = c("NDC", "Mi2", "TIF1γ", "irMyositis"))
conf = y$samples$cartridgeID
conf1 = y$samples$CENTER.BIOPSY
conf2 = y$samples$Conservation
conf = str_remove(conf, "\\s")
conf1 = str_remove(conf1, "\\s")
conf2 = str_remove(conf2, "\\s")
mm <- model.matrix(~0 + group + conf + conf2)


if (any(table(y$samples$Patient) > 1)) {

    voom1 = voom(calcNormFactors(y, method = "TMM"), design = mm, normalize.method = "none", plot = F)

    cons.cor = duplicateCorrelation(voom1, mm, block = factor(y$samples$Patient))$consensus.correlation

    d0 = voom(calcNormFactors(y, method = "TMM"), design = mm, normalize.method = "none", plot = T, block = factor(y$samples$Patient),
        correlation = cons.cor)

} else {

    d0 = voom(calcNormFactors(y, method = "TMM"), design = mm, normalize.method = "none", plot = T)
}
```

```
boxplot(d0$E, ann = F, names = NA)
```

```
dev.off()
```

```
# null device 
#           1
```

```
snames <- colnames(counts)
```


```
fit <- lmFit(d0, mm)
contrlev = paste0("group", c("NDC", "Mi2", "TIF1γ", "irMyositis"))
contrs = combn(colnames(coef(fit))[grepl("group", colnames(coef(fit)))], 2)
contrs = apply(contrs, 2, function(v) {
    ord = match(v, contrlev)
    if (ord[1] < ord[2]) {
        return(v)
    }
    else {
        return(v[c(2, 1)])
    }
})

print(matrix(t(contrs[c(2, 1), ]), ncol = nrow(contrs), nrow = ncol(contrs), dimnames = list(seq(1, ncol(contrs)), c("Case",
    "Reference"))))
```

```
#   Case              Reference   
# 1 "groupMi2"        "groupNDC"  
# 2 "groupTIF1γ"      "groupNDC"  
# 3 "groupirMyositis" "groupNDC"  
# 4 "groupTIF1γ"      "groupMi2"  
# 5 "groupirMyositis" "groupMi2"  
# 6 "groupirMyositis" "groupTIF1γ"
```

```
contrs = paste(apply(t(contrs[c(2, 1), ]), 1, paste, collapse = " - ", sep = " "), collapse = ", ")
comptag = strsplit(contrs, ", ", perl = T)[[1]]
comptag = str_remove_all(comptag, "group")
contrs = strsplit(contrs, ", ", perl = T)[[1]]
contrs = paste(contrs, collapse = ", ")
command = paste("makeContrasts(", contrs, ",levels=mm)", sep = "")
contr = eval(parse(text = command))


tmp <- contrasts.fit(fit, contr)
tmp <- eBayes(tmp, trend = F, robust = F)
addWorksheet(wb, "Moderated t-test,SD trend", gridLines = F)
plotSA(tmp)
```

```
insertPlot(wb, "Moderated t-test,SD trend", width = 8, height = 8, fileType = "png")
top.table <- topTable(tmp, sort.by = "F", n = Inf)
top.table_irmyo = top.table
colnames(top.table)[match(make.names(comptag), str_remove_all(colnames(top.table), "group"))] = comptag
posthoc = as.matrix(decideTests(tmp, method = "hierarchical", adjust.method = "BH", p.value = 0.05))
addWorksheet(wb, "contrast matrix", gridLines = T)
writeDataTable(wb, "contrast matrix", as.data.frame(contr), rowNames = T)
addWorksheet(wb, "Significant genes - F statistic", gridLines = T)
writeDataTable(wb, "Significant genes - F statistic", top.table, rowNames = T)
print(paste("how many significantly DE genes (F statistics, 5% FDR)?", length(which(top.table$adj.P.Val < 0.05))))
```

```
# [1] "how many significantly DE genes (F statistics, 5% FDR)? 93"
```

```
print(paste("how many significant, pairwise tests (post-hoc moderated t-test, 5% FDR)?", sum(abs(posthoc))))
```

```
# [1] "how many significant, pairwise tests (post-hoc moderated t-test, 5% FDR)? 221"
```

```
posttop.table = top.table
posttop.table[, match(str_remove_all(colnames(posthoc), "group"), colnames(posttop.table))] = posttop.table[, match(str_remove_all(colnames(posthoc),
    "group"), colnames(posttop.table))] * abs(posthoc)[rownames(posttop.table), ]
addWorksheet(wb, "DGE - Post Hoc Decide Tests", gridLines = T)
writeDataTable(wb, "DGE - Post Hoc Decide Tests", posttop.table, rowNames = T)

posttop.table[, sapply(posttop.table, is.numeric)] = apply(posttop.table[, sapply(posttop.table, is.numeric)], 2, function(v) (trimws(format(v,
    digits = 2))))


DT::datatable(as.data.frame(posttop.table), caption = "DGE results (post hoc 5% FDR correction)", rownames = F, escape = F,
    extensions = c("FixedColumns", "FixedHeader"), options = list(scrollX = F, paging = T, fixedHeader = T))
```

```
volcanoplots = list()
if (sum(abs(posthoc)) != 0) {
    for (i in seq(1, ncol(posthoc))) {
        test = colnames(posthoc)[i]
        not = colnames(posthoc)[-i]
        get = topTable(tmp, coef = i, number = Inf, adjust.method = "BH")
        getlabs = rownames(get[rownames(posthoc)[which(posthoc[, test] != 0)], ])
        updeg = nrow(get[rownames(posthoc)[which(posthoc[, test] > 0)], ])
        dodeg = nrow(get[rownames(posthoc)[which(posthoc[, test] < 0)], ])
        print(sprintf(fmt = "%i DE genes for comparison %s, %i upregulated and %i downregulated", length(getlabs), test,
            updeg, dodeg))
        get$adj.P.Val = sapply(seq_along(rownames(get)), function(i) {
            g = rownames(get)[i]
            if (g %in% getlabs) {
                return(get[i, , drop = F]$adj.P.Val)
            }
            else {
                return(max(get[i, , drop = F]$adj.P.Val, 0.051))
            }
        })
        getlabs = names(which(sort(abs(setNames(get[getlabs, ]$logFC, getlabs)), decreasing = T)[1:10] > 1))
        if (nrow(get) > 0) {
            colnames(get)[grepl(test, colnames(get))] = paste("LFC(", test, ")", sep = "")
            addWorksheet(wb, test, gridLines = T)
            writeDataTable(wb, test, get, rowNames = T)
            vpname = abbreviate(paste("VP, ", test, sep = ""), minlength = 31)
            addWorksheet(wb, vpname, gridLines = FALSE)
            ggp = EnhancedVolcano(get, title = str_remove_all(test, "group"), lab = rownames(get), selectLab = getlabs, pointSize = 1,
                col = c("wheat3", "wheat3", "maroon3", "maroon3"), legendLabels = c("Not Significant", "Not Significant",
                  "Significant", "Significant"), subtitle = NULL, caption = NULL, labSize = 4, colAlpha = 1, xlab = expression(~log[2] ~
                  "fold-change"), ylab = expression(~-log[10] ~ "adj." ~ "p-value"), max.overlaps = length(getlabs) + 1,
                arrowheads = F, drawConnectors = T, x = "logFC", y = "adj.P.Val", pCutoff = 0.05, FCcutoff = NA, xlim = c(floor(min(get$logFC)),
                  ceiling(max(get$logFC))), ylim = c(0, ceiling(max(-log10(get$adj.P.Val)))))
            print(ggp)
            insertPlot(wb, vpname, width = 10, height = 12, fileType = "png", units = "in")
            volcanoplots[[sprintf(test)]] = ggp
        }
    }
} else {
    print("posthoc indicates no comparison-wise differential expression - no volcano plot will be generated")
}
```

```
# [1] "54 DE genes for comparison groupMi2 - groupNDC, 24 upregulated and 30 downregulated"
```

```
# [1] "55 DE genes for comparison groupTIF1γ - groupNDC, 27 upregulated and 28 downregulated"
```

```
# [1] "35 DE genes for comparison groupirMyositis - groupNDC, 17 upregulated and 18 downregulated"
```

```
# [1] "0 DE genes for comparison groupTIF1γ - groupMi2, 0 upregulated and 0 downregulated"
```

```
# [1] "43 DE genes for comparison groupirMyositis - groupMi2, 19 upregulated and 24 downregulated"
```

```
# [1] "34 DE genes for comparison groupirMyositis - groupTIF1γ, 11 upregulated and 23 downregulated"
```

## 3.2 Post-correction PCA

## 3.3 Gene Set Enrichment Analysis (GSEA)

```
top.rank = top.table[, grepl("-", colnames(top.table))]
colnames(top.rank) = colnames(posthoc)
colnames(top.rank) = str_replace_all(str_replace_all(colnames(posthoc), "group", ""), " - ", "_vs_")


rankgenes = list()
for (i in colnames(top.rank)) {
    rankgenes[[make.names(i)]] = sort(setNames(top.rank[, i, drop = T], rownames(top.rank)), decreasing = T)
}


RCT_ALL_PATHS_GS_and_Toplvl <- read.csv("~/mount-srv1/musellla/Genomic_References/benchmarking_pathways/RCT_ALL_PATHS_GS_and_Toplvl_18022025.csv",
    stringsAsFactors = F)

rct_gs = tapply(RCT_ALL_PATHS_GS_and_Toplvl$GENE, factor(RCT_ALL_PATHS_GS_and_Toplvl$PATH_NAME), function(v) (v))

rct_gs = lapply(rct_gs, function(gs) {

    gs = stringr::str_remove_all(gs, "^[[:alnum:]\\s]+\\:[-_\\.[:alnum:]]+\\s")
    gs = stringr::str_remove_all(gs, "\\s[gG]ene$|\\ssnRNA$|\\s?\\(.+\\)$|\\sRNA\\s")
    gs = gs[!is.na(gs)]
    gs = unique(gs)
    return(gs)
})


rct_gs = rct_gs[!(is.na(rct_gs))]
rct_df = data.frame(TERM = unlist(sapply(names(rct_gs), function(n) (rep(n, length(rct_gs[[n]]))))), GENE = unlist(rct_gs))


aliases = read.delim("~/mount-srv1/musellla/Genomic_References/benchmarking_pathways/9606.protein.aliases.v12.0.txt.gz")

rct2string = aliases$X.string_protein_id[match(rct_df$GENE, aliases$alias)]

ns2string = setNames(aliases$X.string_protein_id[match(setdiff(rownames(top.table), rct_df$GENE), aliases$alias)], setdiff(rownames(top.table),
    rct_df$GENE))

ns2string = ns2string[!is.na(ns2string)]

rct2replace = names(ns2string)[match(rct2string, ns2string)]

rct_df$GENE = ifelse(is.na(rct2replace), rct_df$GENE, rct2replace)


fgs = rownames(top.table[top.table$adj.P.Val <= 0.05, ])


gseCOMPGO = clusterProfiler::compareCluster(geneClusters = rankgenes, fun = "GSEA", TERM2GENE = rct_df, pvalueCutoff = 1,
    verbose = TRUE, eps = 0, minGSSize = 3, maxGSSize = 100, pAdjustMethod = "BH", by = "fgsea", seed = 2202)


gseCOMPGO@compareClusterResult$p.adjust = p.adjust(gseCOMPGO@compareClusterResult$pvalue, "BH")

padjsep = tapply(seq(nrow(gseCOMPGO@compareClusterResult)), factor(gseCOMPGO@compareClusterResult$Cluster), function(i) (setNames(p.adjust(gseCOMPGO@compareClusterResult[i,
    ]$pvalue, "BH"), i)))

names(padjsep) = NULL

padjsep = unlist(padjsep)

gseCOMPGO@compareClusterResult$p.adjust.separate = unname(padjsep)[order(as.numeric(names(padjsep)))]

gseCOMPGO@compareClusterResult = gseCOMPGO@compareClusterResult[gseCOMPGO@compareClusterResult$p.adjust <= 0.05 & gseCOMPGO@compareClusterResult$p.adjust.separate <=
    0.05, ]

gseCOMPGO@compareClusterResult = gseCOMPGO@compareClusterResult[order(abs(gseCOMPGO@compareClusterResult$NES), decreasing = T),
    ]
gseCOMPGO@compareClusterResult = gseCOMPGO@compareClusterResult[order(gseCOMPGO@compareClusterResult$ID), ]
gseCOMP = enrichplot::pairwise_termsim(gseCOMPGO)
gseCOMP@compareClusterResult$Cluster = str_replace_all(gseCOMP@compareClusterResult$Cluster, pattern = "_vs_", replacement = " - ")


gsea2tab = gseCOMP@compareClusterResult

gsea2tab[, sapply(gsea2tab, is.numeric)] = apply(gsea2tab[, sapply(gsea2tab, is.numeric)], 2, format, digits = 2)

DT::datatable(as.data.frame(gsea2tab), caption = "GSEA results (5% FDR)", rownames = F, escape = F, extensions = c("FixedColumns",
    "FixedHeader"), options = list(scrollX = F, paging = T, fixedHeader = T, lengthMenu = list(c(10, 30, 50, -1), c("10",
    "30", "50", "All"))))
```

```
cluslevels = unique(as.character(c(unique(gseCOMP@compareClusterResult$Cluster)[grepl("\\-\\sControl$", unique(gseCOMP@compareClusterResult$Cluster))],
    unique(gseCOMP@compareClusterResult$Cluster)[grepl("^irMyositis\\s\\-", unique(gseCOMP@compareClusterResult$Cluster))],
    unique(gseCOMP@compareClusterResult$Cluster)[!grepl("^irMyositis\\s\\-|\\-\\sControl$", unique(gseCOMP@compareClusterResult$Cluster))])))

cluslevels = cluslevels[match(str_remove_all(colnames(posthoc), "group"), cluslevels)]

gseCOMP@compareClusterResult$Cluster = factor(gseCOMP@compareClusterResult$Cluster, levels = cluslevels)


p1 = enrichplot::dotplot(gseCOMP, showCategory = 8, color = "NES", label_format = 45, split = NULL, font.size = 8, by = "count") +
    scale_color_gradient2(low = "dodgerblue2", high = "violetred2", mid = "white", guide = "colourbar") + theme(rect = element_rect(fill = "transparent"),
    axis.text.x = element_blank(), axis.title.x = element_blank(), axis.ticks.x = element_blank(), axis.text.y = element_text(size = 7,
        face = "bold"), legend.text = element_text(size = 8), legend.position = "top", legend.text.align = 0, legend.title = element_text(size = 8,
        face = "bold"), strip.text = element_text(face = "bold", size = 6.4), strip.background = element_rect(fill = "transparent"),
    legend.direction = "horizontal", legend.justification = "left", legend.key.size = unit(8, "pt")) + xlab("Comparison") +
    scale_y_discrete(labels = function(x) str_wrap(x, width = 40)) + facet_grid(cols = vars(factor(str_replace_all(Cluster,
    "_vs_", " - "), levels = levels(Cluster))), scales = "free_x", space = "free_x", shrink = T, labeller = label_wrap_gen(5))

gseCOMP2plot = gseCOMP

gseCOMP2plot@compareClusterResult$ID = stringr::str_wrap(gseCOMP2plot@compareClusterResult$ID, 30)
gseCOMP2plot@compareClusterResult$Description = stringr::str_wrap(gseCOMP2plot@compareClusterResult$Description, 30)

gseCOMP2plot = enrichplot::pairwise_termsim(gseCOMP2plot)

set.seed(2)

compcols = setNames(randomcoloR::distinctColorPalette(k = length(unique(gseCOMP@compareClusterResult$Cluster)), runTsne = T),
    levels(gseCOMP2plot@compareClusterResult$Cluster))

set.seed(3)

p2 = enrichplot::emapplot(gseCOMP2plot, showCategory = 8, ellipse_style = "ggforce", pie = "count", cex_label_group = 1.4,
    cex_label_category = 0.9, size_line = 0.3, group_category = T, group_legend = F, alpha = 0, direction = "both", type = "t",
    size_category = 1, force = 3, label_group_style = "shadowtext", repel = T, max.overlaps = 20, layout = "nicely", clusterFunction = cluster::fanny) +
    ggtitle(NULL) + scale_color_manual(values = rep("transparent", nrow(gseCOMP@compareClusterResult))) + scale_fill_manual(values = unname(compcols)[order(match(names(compcols),
    levels(gseCOMP@compareClusterResult$Cluster)))], breaks = names(compcols)[order(match(names(compcols), levels(gseCOMP@compareClusterResult$Cluster)))]) +
    labs(fill = "GSEA") + theme(text = element_text(size = 8, family = "sans"), rect = element_rect(color = "transparent",
    fill = "transparent"), legend.text = element_text(size = 8), legend.direction = "horizontal", legend.byrow = T, legend.text.align = 0,
    legend.text.position = "right", legend.title = element_text(size = 8, face = "bold"), legend.position = "top", legend.title.position = "left",
    legend.justification = "left", legend.key.size = unit(8, "pt"), plot.margin = unit(c(0, 0, 0, 0), "cm")) + scale_size_continuous(range = c(2,
    5))
addWorksheet(wb, "GSEA - Dot ~ Concept Network", gridLines = FALSE)
cowplot::plot_grid(p1, p2, ncol = 2, align = "none", rel_widths = c(3, 2), rel_heights = c(1, 1), hjust = -0.3, greedy = T,
    label_size = 10) + theme(legend.key.size = unit(8, "pt"))
```

```
ggsave(filename = paste("Myositis_Nanostring_GSEA_Reactome.svg", sep = ""), width = 10, height = 6)
insertPlot(wb, "GSEA - Dot ~ Concept Network", width = 12, height = 8, fileType = "png")
addWorksheet(wb, "GSEA_PathwayTable", gridLines = T)
writeDataTable(wb, "GSEA_PathwayTable", gseCOMP@compareClusterResult, rowNames = T)
```

# 4 Inspect core enrichment of genes

```
corenrichXcomparison = tapply(gseCOMP@compareClusterResult$core_enrichment, gseCOMP@compareClusterResult$Cluster, function(v) (unique(unlist(sapply(v,
    str_split_1, pattern = "\\/")))))

corenrichXcomparison = lapply(corenrichXcomparison, function(v) (v[top.table[match(v, rownames(top.table)), ]$adj.P.Val <=
    0.05]))
corenrichXcomparison = corenrichXcomparison[sapply(corenrichXcomparison, length) > 0]
allcoreenrich = unique(unlist(corenrichXcomparison))

gene_enrich_ann = data.frame(row.names = allcoreenrich, lapply(corenrichXcomparison, function(v) (ifelse(allcoreenrich %in%
    v, "Yes", "No"))), check.names = F)

gene_enrich_ann = gene_enrich_ann[, match(str_remove_all(colnames(posthoc), "group"), colnames(gene_enrich_ann), nomatch = 0)]


gseas = gseCOMP@compareClusterResult

gseamelt = lapply(gseas$core_enrichment, function(core) {
    d = gseas[grepl(core, gseas$core_enrichment, fixed = T, perl = F), , drop = F]
    gns = data.frame(GENE = str_split(core, "\\/", simplify = F)[[1]])
    return(cbind(gns, do.call("rbind", replicate(nrow(gns), d, simplify = FALSE))))
})

gseamelt = bind_rows(gseamelt)

gseamelt = gseamelt[gseamelt$GENE %in% rownames(gene_enrich_ann), ]


topp = tapply(setNames(gseamelt$NES, gseamelt$ID), factor(gseamelt$GENE), function(v) (names(sort(abs(v[!duplicated(names(v))]))[1])))

topp = unique(unlist(topp))


gseamelt = gseamelt[gseamelt$Description %in% topp, ]

bottom_annotationha = tapply(gseamelt$GENE, factor(gseamelt$Description), function(v) (v))


mat1 = top.table[match(rownames(gene_enrich_ann), rownames(top.table)), ]
mat1 = mat1[, grepl("-", colnames(mat1))]
paletteLength <- 8
myBreaks <- c(seq(min(mat1), 0, length.out = ceiling(paletteLength/2) + 1), seq(max(mat1)/paletteLength, max(mat1), length.out = floor(paletteLength/2)))


colnames(gene_enrich_ann) = paste(colnames(gene_enrich_ann), "(GSEA)")

anncolors = lapply(compcols, function(v) (setNames(c(unname(v), "white"), c("Yes", "No"))))

names(anncolors) = paste(names(anncolors), "(GSEA)")

myColor <- colorRampPalette(c("dodgerblue2", "white", "violetred2"))(8)

colnames(mat1) = paste0(colnames(mat1), " (log2 FC)")


gene_enrich_ann2ha = as.list(gene_enrich_ann)

gene_enrich_ann2ha = as.matrix(as.data.frame(lapply(setNames(nm = names(gene_enrich_ann2ha)), function(n) (ifelse(gene_enrich_ann2ha[[n]] ==
    "Yes", n, "No"))), row.names = rownames(gene_enrich_ann), check.names = F))

colnames(gene_enrich_ann2ha) = NULL

anncolors2ha = do.call("c", lapply(names(anncolors), function(n) (setNames(anncolors[[n]], ifelse(names(anncolors[[n]]) ==
    "Yes", n, "No")))))

anncolors2ha = anncolors2ha[!duplicated(anncolors2ha)]

column_ha = HeatmapAnnotation(`GSEA\nCore Enrichment` = gene_enrich_ann2ha, col = list(`GSEA\nCore Enrichment` = anncolors2ha),
    show_legend = T, which = "column", gp = gpar(col = "grey30", fontsize = 7.2, fontface = "bold"), show_annotation_name = T,
    annotation_name_gp = gpar(fontsize = 7.2, fontface = "bold"), height = unit(0.8, "cm"), simple_anno_size_adjust = T,
    gap = unit(0, "npc"))


column2ann = lapply(bottom_annotationha, function(v) (ifelse(is.na(match(rownames(gene_enrich_ann), v)), "white", "black")))

column2_ha = HeatmapAnnotation(df = as.data.frame(column2ann, check.names = F), col = lapply(column2ann, function(v) (setNames(nm = unique(v)))),
    show_legend = F, which = "column", gp = gpar(col = "gray90", fontsize = 7.2, fontface = "bold"), height = unit(3.8, "cm"),
    gap = unit(0.5, "mm"), simple_anno_size_adjust = T, annotation_name_gp = gpar(fontsize = 7.2, fontface = 2))


row_ha = HeatmapAnnotation(DGE = names(compcols), col = list(DGE = compcols), which = "row", gp = gpar(col = "gray30"), show_annotation_name = T,
    show_legend = T, annotation_name_gp = gpar(fontsize = 7.2, fontface = "bold"), width = unit(2, "mm"), annotation_legend_param = list(DGE = list(legend_direction = "horizontal")),
    simple_anno_size_adjust = T)


Heatmap(t(mat1)[colnames(mat1), ], row_names_gp = gpar(col = NA), show_heatmap_legend = F, column_names_gp = gpar(fontsize = 6.1,
    fontface = 1), col = myColor, heatmap_legend_param = list(title = expression(bold(log[2] ~ "Fold-change")), title_gp = gpar(fontsize = 7.5,
    fontface = 1), grid_height = unit(3, "mm"), grid_width = unit(3, "mm"), legend_width = unit(27, "mm"), labels_rot = 45,
    at = sort(unique(c(seq(min(mat1), 0, length.out = 3), seq(0, max(mat1), length.out = 3)))), labels = c(signif(sort(unique(c(seq(min(mat1),
        0, length.out = 3), seq(0, max(mat1), length.out = 3)))), 2)), legend_direction = "horizontal", legend_position = "bottom",
    labels_gp = gpar(fontsize = 7)), cluster_columns = as.hclust(reorder(as.dendrogram(hclust(dist(mat1))), wts = match(rownames(gene_enrich_ann[do.call(order,
    c(gene_enrich_ann, decreasing = T)), ]), hclust(dist(mat1))$labels))), cluster_rows = as.hclust(reorder(as.dendrogram(hclust(dist(t(mat1)))),
    wts = match(str_remove_all(colnames(gene_enrich_ann), "\\s+GSEA"), hclust(dist(t(mat1)))$labels))), top_annotation = c(column_ha,
    column2_ha), left_annotation = row_ha, border_gp = gpar(col = "gray30", lwd = 0.5), rect_gp = gpar(col = "gray45", lwd = 0.5),
    show_column_dend = F, show_row_dend = F, column_names_rot = -45)
```

## 4.1 BoxPlots for each F-statistics-significant gene in a GSEA core-enrichment

```
batchcorrenrich = removeBatchEffect(d0, batch = conf, batch2 = conf2)
batchcorrenrich = batchcorrenrich[match(allcoreenrich, rownames(batchcorrenrich)), ]
explst = lapply(as.data.frame(t(batchcorrenrich)), function(exps) (setNames(exps, colnames(batchcorrenrich))))
boxdotdfs = lapply(explst, function(exps) (df = data.frame(HKnormalizedCounts = unname(exps), Subtype = sampinf$Subtype[match(names(exps),
    sampinf$Biomarker.Nr.)], row.names = names(exps), check.names = F, check.rows = F)))
boxdotdfs = cbind(bind_rows(boxdotdfs), data.frame(Gene = rep(names(boxdotdfs), each = ncol(batchcorrenrich))))
boxdotdfs$Subtype = factor(boxdotdfs$Subtype, levels = c("NDC", "Mi2", "TIF1γ", "irMyositis"))
boxdotdfs$Gene = factor(boxdotdfs$Gene, levels = as.hclust(reorder(as.dendrogram(hclust(dist(mat1))), wts = match(rownames(gene_enrich_ann[do.call(order,
    c(gene_enrich_ann, decreasing = T)), ]), hclust(dist(mat1))$labels)))$labels)


genebyexp = names(sort(tapply(boxdotdfs$HKnormalizedCounts, factor(boxdotdfs$Gene), mean)))

sort_index <- match(boxdotdfs$Gene, genebyexp)

boxdotdfs = boxdotdfs[order(sort_index), ]

boxdotdfs$Gene = factor(boxdotdfs$Gene, levels = genebyexp)


stat.res = as.data.frame(posthoc[levels(boxdotdfs$Gene), ])
stat.res = lapply(as.data.frame(t(stat.res)), function(v) (setNames(v, str_remove_all(colnames(stat.res), "group"))))
stat.res = lapply(stat.res, function(v) (setNames(rep(0.05, length(v[v != 0])), names(v[v != 0]))))


mspg = max(sapply(stat.res, length))

annotation_df = data.frame(Gene = factor(unname(unlist(mapply(function(x, y) (rep(x, y)), names(stat.res), sapply(stat.res,
    length)))), levels = levels(boxdotdfs$Gene)), Treatment = factor(str_match(unname(unlist(sapply(stat.res, names))), "(^.+)\\s-\\s")[,
    2], levels = levels(boxdotdfs$Subtype)), Control = factor(str_match(unname(unlist(sapply(stat.res, names))), "^.+\\s-\\s(.+$)")[,
    2], levels = levels(boxdotdfs$Subtype)), Ypos = unname(unlist(mapply(function(x, y) (rep(x, y)), unname(tapply(boxdotdfs$HKnormalizedCounts,
    boxdotdfs$Gene, max)) + 0.7, sapply(stat.res, length)))) + unlist(unname(sapply(sapply(stat.res, length)[sapply(stat.res,
    length) > 0], function(v) (seq(0, 0.3 * mspg, by = 0.3)[1:v])))), Pvalues = "")
annotation_df = with(annotation_df, annotation_df[order(Gene, Treatment, Control), ])
annotation_df$Ypos = unlist(tapply(annotation_df$Ypos, annotation_df$Gene, sort))


ggplot(boxdotdfs, aes(y = HKnormalizedCounts, x = Subtype, fill = Subtype)) + geom_boxplot(outlier.shape = NA, notch = F,
    alpha = 1) + geom_jitter(aes(y = HKnormalizedCounts, x = Subtype, fill = stage(Subtype, after_scale = alpha(fill, 0.2))),
    color = "black", shape = 21, stroke = 0.4, position = position_dodge2(width = 0.25), size = 1, inherit.aes = F) + theme_classic(base_size = 10) +
    labs(y = expression(bold("Batch-corrected" ~ log["2"] ~ "CPM"))) + theme(text = element_text(color = "black", face = "bold"),
    axis.text.x = element_blank(), axis.text.y = element_text(color = "black", size = 8, face = "bold"), axis.ticks.x = element_blank(),
    axis.line.x = element_blank(), axis.title.x = element_blank(), axis.title.y = element_text(face = "bold"), strip.text = element_text(size = 7),
    legend.text = element_text(size = 10), legend.direction = "horizontal", legend.position = "top") + scale_fill_manual(values = c("lemonchiffon3",
    "orange3", "maroon3", "turquoise3")) + geom_signif(data = annotation_df, aes(xmin = Control, xmax = Treatment, annotations = Pvalues,
    y_position = Ypos), vjust = -0.2, textsize = 4, tip_length = 0.01, manual = TRUE, inherit.aes = F, show.legend = F) +
    facet_wrap(~Gene, ncol = 11, as.table = T)
```

# 5 Inspect DGE of genes related to Interferon signalling

```
POIs = names(which(table(gseCOMPGO@compareClusterResult$ID) > 1))

POIs = POIs[grepl("Antiviral|Interferon|DDX58", POIs, ignore.case = T)]

print(POIs)
```

```
# [1] "Antiviral mechanism by IFN-stimulated genes"  "Interferon alpha/beta signaling"             
# [3] "Interferon Signaling"                         "ISG15 antiviral mechanism"                   
# [5] "Negative regulators of DDX58/IFIH1 signaling"
```

```
INFABgenes = rct_df[grepl(paste(POIs, sep = "|", collapse = "|"), rct_df$TERM, ignore.case = T), ]
INFABcorenrich = c(unique(unlist(sapply(gseCOMP@compareClusterResult$core_enrichment[grepl("^Interferon.+[Ss]ignaling$|IFN",
    gseCOMP@compareClusterResult$ID)], str_split_1, pattern = "\\/"))), rownames(top.table)[grepl("^IFN.*", rownames(top.table))])


ifngenes = INFABgenes[INFABgenes$GENE %in% rownames(top.table), ]$GENE

INFABgenes = INFABgenes[INFABgenes$GENE %in% INFABcorenrich, ]
INFABgenes = INFABgenes[!duplicated(INFABgenes), ]
gene2path = tapply(INFABgenes$GENE, factor(INFABgenes$TERM), function(v) (v))
gene_ann = data.frame(row.names = unique(INFABgenes$GENE), `Type II Interferon Signalling` = ifelse(unique(INFABgenes$GENE) %in%
    gene2path$`Interferon gamma signaling`, "Yes", "No"), `Type I Interferon Signalling` = ifelse(unique(INFABgenes$GENE) %in%
    gene2path$`Interferon alpha/beta signaling`, "Yes", "No"), `DGE (5% FDR F-statistics)` = ifelse(top.table[match(unique(INFABgenes$GENE),
    rownames(top.table)), ]$adj.P.Val <= 0.05, "Significant", "Not Significant"), check.names = F)


minlogpval = -log10(min(top.table$adj.P.Val)) + 0.5
minlfc = min(top.table[, grepl("-", colnames(top.table))]) - 0.5
maxlfc = max(top.table[, grepl("-", colnames(top.table))]) + 0.5

if (sum(abs(posthoc)) != 0) {
    for (i in seq(1, ncol(posthoc))) {
        test = colnames(posthoc)[i]
        not = colnames(posthoc)[-i]
        get = topTable(tmp, coef = i, number = Inf, adjust.method = "BH")
        minip = -log10(min(get$adj.P.Val))
        if (minlogpval < minip) {
            minlogpval = ceiling(minip)
        }
    }
}

DGEandIFN = intersect(INFABgenes$GENE, rownames(posttop.table))

IFNPostHocDF = merge(INFABgenes[INFABgenes$GENE %in% DGEandIFN, ], cbind(data.frame(GENE = rownames(posttop.table)), posttop.table[,
    -c(1, 2, 3)])[match(DGEandIFN, rownames(posttop.table)), ], by = "GENE", all.x = T, all.y = T, no.dups = F)

IFNPostHocDF = IFNPostHocDF[!apply(IFNPostHocDF[, grepl(" - ", colnames(IFNPostHocDF))], 1, function(v) (all(as.numeric(v) ==
    0))), ]

DT::datatable(as.data.frame(IFNPostHocDF), caption = "DGE of interferon-related genes (post hoc 5% FDR correction)", rownames = F,
    escape = F, extensions = c("FixedColumns", "FixedHeader"), options = list(scrollX = F, paging = T, fixedHeader = T))
```

```
addWorksheet(wb, "DGE post hoc Interferon-related", gridLines = T)
writeDataTable(wb, "DGE post hoc Interferon-related", as.data.frame(IFNPostHocDF), rowNames = T)

volcanoplots = list()
if (sum(abs(posthoc)) != 0) {
    for (i in seq(1, ncol(posthoc))) {
        test = colnames(posthoc)[i]
        not = colnames(posthoc)[-i]
        get = topTable(tmp, coef = i, number = Inf, adjust.method = "BH")
        getlabs = rownames(get[rownames(posthoc)[which(posthoc[, test] != 0)], ])
        updeg = nrow(get[rownames(posthoc)[which(posthoc[, test] > 0)], ])
        dodeg = nrow(get[rownames(posthoc)[which(posthoc[, test] < 0)], ])
        print(sprintf(fmt = "%i DE genes for comparison %s, %i upregulated and %i downregulated", length(getlabs), test,
            updeg, dodeg))
        get$adj.P.Val = sapply(seq_along(rownames(get)), function(i) {
            g = rownames(get)[i]
            if (g %in% getlabs) {
                return(get[i, , drop = F]$adj.P.Val)
            }
            else {
                return(max(get[i, , drop = F]$adj.P.Val, 0.051))
            }
        })
        getlabs = intersect(getlabs, INFABgenes$GENE)
        if (nrow(get) > 0) {
            colnames(get)[grepl(test, colnames(get))] = paste("LFC(", test, ")", sep = "")
            vpname = abbreviate(paste("VP, ", test, sep = ""), minlength = 31)

            ggp = EnhancedVolcano(get, title = str_remove_all(test, "group"), lab = rownames(get), borderColour = "black",
                titleLabSize = 10, pointSize = 1, selectLab = getlabs, labFace = "bold", col = c("antiquewhite3", "antiquewhite3",
                  "violetred3", "violetred3"), legendLabels = c("Not Significant", "Not Significant", "Significant (5% FDR)",
                  "Significant (5% FDR)"), subtitle = NULL, caption = NULL, labSize = 2.2, colAlpha = 0.5, xlab = expression(~log[2] ~
                  "fold-change"), ylab = expression(~-log[10] ~ "BH-adjusted" ~ "p-value"), max.overlaps = length(getlabs) +
                  20, arrowheads = F, drawConnectors = T, widthConnectors = 0.2, x = "logFC", y = "adj.P.Val", pCutoff = 0.05,
                FCcutoff = NA, xlim = c(minlfc, maxlfc), ylim = c(0, minlogpval)) + ggplot2::theme(legend.text = element_text(color = "black",
                size = 10), axis.text.x = element_text(color = "black", size = 10), axis.text.y = element_text(color = "black",
                size = 10), axis.text.r = element_text(color = "black", size = 10), axis.title.x = element_text(color = "black",
                size = 12, face = "bold"), axis.title.y = element_text(color = "black", size = 12, face = "bold"), panel.grid.major = element_line(linewidth = 0.04,
                color = "gray60"), panel.grid.minor = element_blank())
            if (i %in% 1:3) {
                ggp = ggp + rremove("xlab")
            }
            if (!(i %in% c(1, 4))) {
                ggp = ggp + rremove("ylab")
            }

            volcanoplots[[sprintf(test)]] = ggp
        }
    }
} else {
    print("posthoc indicates no comparison-wise differential expression - no volcano plot will be generated")
}
```

```
# [1] "54 DE genes for comparison groupMi2 - groupNDC, 24 upregulated and 30 downregulated"
# [1] "55 DE genes for comparison groupTIF1γ - groupNDC, 27 upregulated and 28 downregulated"
# [1] "35 DE genes for comparison groupirMyositis - groupNDC, 17 upregulated and 18 downregulated"
# [1] "0 DE genes for comparison groupTIF1γ - groupMi2, 0 upregulated and 0 downregulated"
# [1] "43 DE genes for comparison groupirMyositis - groupMi2, 19 upregulated and 24 downregulated"
# [1] "34 DE genes for comparison groupirMyositis - groupTIF1γ, 11 upregulated and 23 downregulated"
```

```
combined_plot <- patchwork::wrap_plots(volcanoplots, ncol = 3, nrow = 2) + patchwork::plot_layout(guides = "collect") & theme(legend.position = "bottom")

print(combined_plot)
```


```
upinir = c(names(which(apply(posthoc[, c(4, 5)], 1, sum) > 1)))

print(upinir)


IFNgraph = readRDS("ALL_PATHS_WITH_Upregulated_irDEGs__AND_PDL1.rds.gz")

print(table(RCT_ALL_PATHS_GS_and_Toplvl[match(E(IFNgraph)$PATHWAY_NAME, RCT_ALL_PATHS_GS_and_Toplvl$PATH_NAME), ]$TOP_LEVEL_PATHWAY))

IFNgraph = delete.edges(IFNgraph, setdiff(seq_along(E(IFNgraph)), which(RCT_ALL_PATHS_GS_and_Toplvl[match(E(IFNgraph)$PATHWAY_NAME,
    RCT_ALL_PATHS_GS_and_Toplvl$PATH_NAME), ]$TOP_LEVEL_PATHWAY %in% c("Disease", "Immune System", "Signal Transduction"))))

IFNgraph = delete.vertices(IFNgraph, which(degree(IFNgraph) == 0))

IFNgraph = igraph::simplify(IFNgraph, edge.attr.comb = "concat")

V(IFNgraph)[grepl("RIGI", V(IFNgraph)$name)]$name = "DDX58"
V(IFNgraph)[grepl("cd21", V(IFNgraph)$name)]$name = "CR2"

diameter(IFNgraph)


irDEGs = c(names(which(apply(abs(posthoc[, 2:5]), 1, sum) != 0)))

nsinnet = intersect(irDEGs, V(IFNgraph)$name)


extract_vertex_info <- function(vs) {
    list(indices = as.vector(vs), names = names(vs))
}

cl = parallel::makeCluster(16, "PSOCK")
parallel::clusterEvalQ(cl, library(igraph))
parallel::clusterExport(cl, c("extract_vertex_info", "nsinnet", "IFNgraph"))


APSPs = parallel::parLapply(cl, combn(nsinnet, 2, simplify = F), function(l) {

    asps = c(all_shortest_paths(IFNgraph, from = l[[1]], to = l[[2]], mode = "out")$res, all_shortest_paths(IFNgraph, from = l[[1]],
        to = l[[2]], mode = "in")$res)

    vertex_info <- lapply(asps, extract_vertex_info)

    asps = asps[duplicated(vertex_info, fromLast = FALSE)]

    return(lapply(asps, function(r) {
        r = names(r)
        lr = length(r)
        x = sapply(seq(length(r)), function(i) {
            if (i == 1 | i == length(r)) {
                return(r[i])
            } else {
                return(rep(r[i], 2))
            }
        })
        return(x)
    }))
})

parallel::stopCluster(cl)

gc()

IFNgraph = delete.edges(IFNgraph, setdiff(seq_along(E(IFNgraph)), unique(get.edge.ids(IFNgraph, unlist(APSPs), directed = F))))

IFNgraph = delete.vertices(IFNgraph, which(degree(IFNgraph) == 0))


ecomps = unlist(sapply(E(IFNgraph)$REGULATION_MODE, function(v) {
    v = unique(unlist(v))
    v = v[v != "null" & v != ""]
    return(paste(unique(sort(v)), collapse = " AND "))
}))

table(ecomps)


pnreg = mapply(FUN = function(x, y) {

    if (x != 0) {
        if (y != 0) {
            return(2)
        }
        else {
            return(1)
        }
    }
    else {
        if (y != 0) {
            return(-1)
        }
        else {
            return(0)
        }
    }
}, str_count(string = ecomps, "Positive"), str_count(string = ecomps, "Negative"))


E(IFNgraph)$eloc = pnreg


E(IFNgraph)$color = c("lightskyblue", "#cbcbcbff", "hotpink1", "purple3")[as.numeric(factor(E(IFNgraph)$eloc))]

V(IFNgraph)$size = Vectorize(function(v) {
    if (v %in% c(rownames(posthoc), bad)) {
        return(5)
    }
    else {
        return(0)
    }
})(V(IFNgraph)$name)
V(IFNgraph)$label = ifelse(V(IFNgraph)$name %in% c(rownames(posthoc), bad), V(IFNgraph)$name, NA)
V(IFNgraph)$label.family = "sans"
V(IFNgraph)$label.dist = Vectorize(function(v) {
    if (v %in% c(rownames(posthoc), bad)) {
        return(0.6)
    }
    else {
        return(0)
    }
})(V(IFNgraph)$name)
V(IFNgraph)$pie.lty = Vectorize(function(v) {
    if (v %in% rownames(posthoc)) {
        return("solid")
    }
    else {
        return("dotted")
    }
})(V(IFNgraph)$name)
V(IFNgraph)$shape = Vectorize(function(v) {
    if (v %in% intersect(V(IFNgraph)$name, rownames(colcountraw)[colcountraw$Code.Class == "Endogenous"])) {
        return("pie")
    }
    else {
        return("none")
    }
})(V(IFNgraph)$name)


COIs = colnames(posthoc)[c(5, 4, 3, 2)][c(3, 1, 2, 4)]
ngroups = length(COIs)
V(IFNgraph)$pie = unname(as.list(Vectorize(function(v) {
    if (v %in% rownames(posthoc)) {
        return(rep(1, ngroups))
    }
    else {
        return(1)
    }
})(V(IFNgraph)$name)))

piecols = c("turquoise2", "white", "maroon2")
V(IFNgraph)$pie.color = unname(as.list(Vectorize(function(v) {
    if (v %in% rownames(posthoc)) {
        dges = as.vector(posthoc[v, COIs]) + 2
        return(piecols[dges])
    }
    else if (v %in% bad) {
        return("white")
    }
    else {
        return("black")
    }
})(V(IFNgraph)$name)))
V(IFNgraph)$pie.border = unname(as.list(Vectorize(function(v) {
    if (v %in% rownames(posthoc)) {
        dges = as.vector(posthoc[v, COIs]) + 2
        return(piecols[dges])
    }
    else if (v %in% bad) {
        return("black")
    }
    else {
        return("black")
    }
})(V(IFNgraph)$name)))
V(IFNgraph)$frame.lty = 3
V(IFNgraph)$frame.color = NA
V(IFNgraph)$avexp = top.table[match(V(IFNgraph)$name, rownames(top.table)), ]$AveExpr
V(IFNgraph)$avexp = as.numeric(ifelse(is.na(V(IFNgraph)$avexp), max(V(IFNgraph)$avexp, na.rm = T), V(IFNgraph)$avexp))
avexppal <- c("lightgoldenrod3", "azure4", "black")
V(IFNgraph)$frame.color = avexppal[as.numeric(cut(V(IFNgraph)$avexp, breaks = quantile(top.table$AveExpr, probs = c(0, 0.25,
    0.75, 1)), include.lowest = T, right = T))]
E(IFNgraph)$width = 0.4
E(IFNgraph)$arrow.size = 0.2
E(IFNgraph)$arrow.width = 0.3
E(IFNgraph)$weight = 0.5
V(IFNgraph)$frame.width = 10


par(mfrow = c(1, 1), mar = c(0, 0, 0, 0), family = "bold")


graph2plot = delete.edges(IFNgraph, which(E(IFNgraph)$color == "white"))
graph2plot = delete.vertices(graph2plot, which(degree(graph2plot) == 0))
V(graph2plot)$label = V(graph2plot)$name
V(graph2plot)$label.font = 2


graph2plot = induced.subgraph(graph2plot, communities(igraph::components(graph2plot))[[which.max(igraph::components(graph2plot)$csize)]])


which(degree(graph2plot) <= 1)


set.seed(1)
l <- qgraph::qgraph.layout.fruchtermanreingold(get.edgelist(graph2plot, names = F), vcount = vcount(graph2plot), niter = 5000,
    area = 10 * (vcount(graph2plot)^1.6), repulse.rad = (vcount(graph2plot)^2), init = layout_components(graph2plot))


GOIs = intersect(c(upinir, "CD274", "PDCD1"), V(graph2plot)$name)


hitpathsout = unlist(lapply(lapply(lapply(GOIs, function(n) (all_shortest_paths(graph2plot, from = n, to = setdiff(GOIs,
    n), mode = "all"))), "[[", "res"), function(res) {
    return(unlist(lapply(res, function(r) {
        r = names(r)
        lr = length(r)
        x = sapply(seq(length(r)), function(i) {
            if (i == 1 | i == length(r)) {
                return(r[i])
            } else {
                return(rep(r[i], 2))
            }
        })
        return(x)
    })))
}))


E(graph2plot)$lty = 3

E(graph2plot)[unique(get.edge.ids(graph2plot, hitpathsout, directed = F))]$lty = 1

E(graph2plot)[unique(get.edge.ids(graph2plot, hitpathsout, directed = F))]$width = 1

plot.new()

plot(graph2plot, layout = l, vertex.frame.width = 10, vertex.label.cex = 0.65, edge.lty = E(graph2plot)$lty, vertex.label.degree = -pi/2,
    vertex.color = "white", vertex.frame.lty = "solid", vertex.label.color = "black")
```

# 6 All irDEGs and ICI targets APSPs

```
irDEGs = unique(c(Reduce(intersect, list(names(which(apply(posthoc[, c(1)], 1, sum) <= 0)), names(which(apply(posthoc[, c(2)],
    1, sum) <= 0)), names(which(apply(posthoc[, c(5:6)], 1, sum) > 1)))), Reduce(intersect, list(names(which(apply(posthoc[,
    c(1)], 1, sum) >= 0)), names(which(apply(posthoc[, c(2)], 1, sum) >= 0)), names(which(apply(posthoc[, c(5:6)], 1, sum) <
    -1))))))


irTable = posttop.table[match(sort(irDEGs), rownames(posttop.table)), ]

DT::datatable(as.data.frame(irTable), caption = "irDEGs and DGE exclusive to irMyositis (post hoc 5% FDR correction)", rownames = F,
    escape = F, extensions = c("FixedColumns", "FixedHeader"), options = list(scrollX = F, paging = T, fixedHeader = T, lengthMenu = list(c(10,
        -1), c("10", "All"))))
```

```
addWorksheet(wb, "irDEGs post hoc Table", gridLines = T)
writeDataTable(wb, "irDEGs post hoc Table", as.data.frame(irTable), rowNames = T)
```

The Reactome interactions were queried using Neo4j/Cypher. The query was the following:

```
MATCH (n:ReferenceEntity)-[:referenceEntity]-(:EntityWithAccessionedSequence{speciesName:"Homo sapiens"})<-[:input|output|catalystActivity|physicalEntity|regulatedBy|regulator|hasComponent|hasMember|hasCandidate*]-(:ReactionLikeEvent{speciesName:"Homo sapiens"})<-[:hasEvent]-(p:Pathway{speciesName:"Homo sapiens", schemaClass:"Pathway"})
WHERE any(x IN n.geneName WHERE x IN [
'BST2',
'CASP10',
'CCL14',
'CFB',
'CR1',
'DDX58',
'FCGR1A',
'FN1',
'IFI16',
'IFI27',
'IFI35',
'IFIH1',
'IFIT1',
'IFIT2',
'IFITM1',
'IFNG',
'IL1RAP',
'IRF7',
'ISG15',
'ISG20',
'KLRC2',
'MX1',
'NT5E',
'OAS3',
'STAT2',
'TGFB2',
'TIGIT',
'TNFRSF8',
'PDCD1',
'CD274',
'CTLA4'
])
unwind [p] as nums
WITH COLLECT(distinct nums) as rles
CALL apoc.cypher.parallel2(
"MATCH (p:Pathway)-[:hasEvent*]->(rle)
OPTIONAL MATCH (rle)-[:compartment]-(comp:Compartment)
OPTIONAL MATCH (rle)-[:catalystActivity]-(:CatalystActivity)-[:activity]-(go:GO_MolecularFunction)
OPTIONAL MATCH (rle)-[:regulatedBy]-(reg:Regulation)-[:regulator|activeUnit|catalystActivity|hasComponent|hasMember|hasCandidate*]->(ereg:EntityWithAccessionedSequence{speciesName:'Homo sapiens'})-[:referenceEntity]-(ref_reg:ReferenceEntity)-[:referenceDatabase]-(rdb_reg:ReferenceDatabase)
MATCH (rle)-[:input|physicalEntity|hasComponent|hasMember|hasCandidate*]->(einp:EntityWithAccessionedSequence{speciesName:'Homo sapiens'})-[:referenceEntity]-(ref_in:ReferenceEntity)-[:referenceDatabase]-(rdb_in:ReferenceDatabase),
(rle)-[:output|physicalEntity|hasComponent|hasMember|hasCandidate*]->(eout:EntityWithAccessionedSequence{speciesName:'Homo sapiens'})-[:referenceEntity]-(ref_out:ReferenceEntity)-[:referenceDatabase]-(rdb_out:ReferenceDatabase)
RETURN DISTINCT p.displayName AS PATHWAY_NAME, p.stId AS PATHWAY_ID, rle.stId AS RLE_ID, rle.displayName AS RLE_NAME, comp.displayName AS COMPARTMENT, rle.category AS RLE_CATEGORY,  ref_in.displayName AS SOURCE, rdb_in.displayName AS SOURCE_DB, ref_out.displayName AS TARGET, einp.displayName AS SOURCE_CC, eout.displayName AS TARGET_CC, reg.schemaClass AS REGULATION_MODE, go.displayName AS REGULATOR_ACTIVITY, ref_reg.displayName AS REGULATOR, ereg.displayName AS REGULATOR_CC, rdb_out.displayName AS TARGET_DB, rdb_reg.displayName AS REGULATOR_DB",
  {p: rles},
  'p')
  YIELD value
RETURN DISTINCT value.PATHWAY_NAME AS PATHWAY_NAME, value.PATHWAY_ID AS PATHWAY_ID, value.RLE_ID AS RLE_ID, value.RLE_NAME AS RLE_NAME, value.COMPARTMENT AS COMPARTMENT, value.RLE_CATEGORY AS RLE_CATEGORY, value.SOURCE AS SOURCE, value.SOURCE_DB AS SOURCE_DB, value.SOURCE_CC AS SOURCE_CC, value.TARGET AS TARGET, value.TARGET_DB AS TARGET_DB, value.TARGET_CC AS TARGET_CC, value.REGULATION_MODE AS REGULATION_MODE, value.REGULATOR_ACTIVITY AS GO_MOLECULAR_FUNCTION, value.REGULATOR AS REGULATOR, value.REGULATOR_DB AS REGULATOR_DB, value.REGULATOR_CC AS REGULATOR_CC
```

```
IFNgraph = readRDS("~/mount-srv1/musellla/code/R/musellla_R_workspace/Colitis_MelAutim/ALL_PATHS_WITH_irDEGs_AND_PDL1_AND_CTLA4.rds.gz")

print(table(RCT_ALL_PATHS_GS_and_Toplvl[match(E(IFNgraph)$PATHWAY_NAME, RCT_ALL_PATHS_GS_and_Toplvl$PATH_NAME), ]$TOP_LEVEL_PATHWAY))
```

```
# 
#                           Disease                        DNA Repair Extracellular matrix organization 
#                            150982                              4024                              8320 
#   Gene expression (Transcription)                        Hemostasis                     Immune System 
#                               377                             12202                            250964 
#            Metabolism of proteins                   Neuronal System               Signal Transduction 
#                             27607                               180                             29418
```

```
IFNgraph = delete.vertices(IFNgraph, which(degree(IFNgraph) == 0))


E(IFNgraph)$EXTRACELLULAR_INPUT = sapply(E(IFNgraph)$COMPARTMENT_PAIRWISE, function(v) (str_count(v, "^\\[extracellular|^\\[[:alnum:\\s]*secret[a-z\\s]*,")))

E(IFNgraph)$EXTRACELLULAR_OUTPUT = sapply(E(IFNgraph)$COMPARTMENT_PAIRWISE, function(v) (str_count(v, ",\\sextracellular\\s[:alnum:]*\\]$|,\\s[:alnum:\\s]*secret[a-z\\s]*\\]$")))

IFNgraph = igraph::simplify(IFNgraph, edge.attr.comb = "concat")


V(IFNgraph)[grepl("RIGI", V(IFNgraph)$name)]$name = "DDX58"
V(IFNgraph)[grepl("cd21", V(IFNgraph)$name)]$name = "CR2"

diameter(IFNgraph)
```

```
# [1] 10
```

```
irDEGs = c(irDEGs, c("CD274", "PDCD1", "CTLA4"))

nsinnet = intersect(irDEGs, V(IFNgraph)$name)


extract_vertex_info <- function(vs) {
    list(indices = as.vector(vs), names = names(vs))
}

cl = parallel::makeCluster(32, "PSOCK")
parallel::clusterExport(cl, c("extract_vertex_info", "nsinnet", "IFNgraph"))

APSPs = parallel::parLapply(cl, combn(nsinnet, 2, simplify = F), function(l) {

    asps = igraph::all_shortest_paths(IFNgraph, from = l[[1]], to = l[[2]], mode = "all")$res

    vertex_info <- lapply(asps, extract_vertex_info)

    asps = asps[duplicated(vertex_info, fromLast = FALSE)]

    return(lapply(asps, function(r) {
        r = names(r)
        lr = length(r)
        x = sapply(seq(length(r)), function(i) {
            if (i == 1 | i == length(r)) {
                return(r[i])
            } else {
                return(rep(r[i], 2))
            }
        })
        return(x)
    }))
})

parallel::stopCluster(cl)

gc()
```

```
#            used  (Mb) gc trigger  (Mb) max used  (Mb)
# Ncells 12118288 647.2   18232620 973.8 18232620 973.8
# Vcells 56232657 429.1  103693346 791.2 89312185 681.4
```

```
IFNgraph = delete.edges(IFNgraph, setdiff(seq_along(E(IFNgraph)), unique(get.edge.ids(IFNgraph, unlist(APSPs), directed = F))))

IFNgraph = delete.vertices(IFNgraph, which(degree(IFNgraph) == 0))


ecomps = unlist(sapply(E(IFNgraph)$REGULATION_MODE, function(v) {
    v = unique(unlist(v))
    v = v[v != "null" & v != ""]
    return(paste(unique(sort(v)), collapse = " AND "))
}))

table(ecomps)
```

```
# ecomps
#                                                                       
#                                                                  1288 
#                                      NegativeGeneExpressionRegulation 
#                                                                     3 
# NegativeGeneExpressionRegulation AND PositiveGeneExpressionRegulation 
#                                                                     4 
#                                                    NegativeRegulation 
#                                                                   159 
#                             NegativeRegulation AND PositiveRegulation 
#                                                                     8 
#                                      PositiveGeneExpressionRegulation 
#                                                                     6 
#               PositiveGeneExpressionRegulation AND PositiveRegulation 
#                                                                     1 
#                                                    PositiveRegulation 
#                                                                   505
```

```
pnreg = mapply(FUN = function(x, y) {

    if (x != 0) {
        if (y != 0) {
            return(2)
        }
        else {
            return(1)
        }
    }
    else {
        if (y != 0) {
            return(-1)
        }
        else {
            return(0)
        }
    }
}, str_count(string = ecomps, "Positive"), str_count(string = ecomps, "Negative"))


E(IFNgraph)$eloc = pnreg


E(IFNgraph)$color = c("lightskyblue", "#cbcbcbff", "hotpink1", "purple3")[as.numeric(factor(E(IFNgraph)$eloc))]

V(IFNgraph)$size = Vectorize(function(v) {
    if (v %in% c(rownames(posthoc), bad)) {
        return(5)
    }
    else {
        return(0)
    }
})(V(IFNgraph)$name)
V(IFNgraph)$label = ifelse(V(IFNgraph)$name %in% c(rownames(posthoc), bad), V(IFNgraph)$name, NA)
V(IFNgraph)$label.family = "sans"
V(IFNgraph)$label.dist = Vectorize(function(v) {
    if (v %in% c(rownames(posthoc), bad)) {
        return(0.6)
    }
    else {
        return(0)
    }
})(V(IFNgraph)$name)
V(IFNgraph)$pie.lty = Vectorize(function(v) {
    if (v %in% rownames(posthoc)) {
        return("solid")
    }
    else {
        return("dotted")
    }
})(V(IFNgraph)$name)
V(IFNgraph)$shape = Vectorize(function(v) {
    if (v %in% intersect(V(IFNgraph)$name, rownames(colcountraw)[colcountraw$Code.Class == "Endogenous"])) {
        return("pie")
    }
    else {
        return("none")
    }
})(V(IFNgraph)$name)


COIs = colnames(posthoc)[c(6, 5, 2, 1)][c(4, 2, 1, 3)]

ngroups = length(COIs)
V(IFNgraph)$pie = unname(as.list(Vectorize(function(v) {
    if (v %in% rownames(posthoc)) {
        return(rep(1, ngroups))
    }
    else {
        return(1)
    }
})(V(IFNgraph)$name)))
piecols = c("turquoise2", "white", "maroon2")
V(IFNgraph)$pie.color = unname(as.list(Vectorize(function(v) {
    if (v %in% rownames(posthoc)) {
        dges = as.vector(posthoc[v, COIs]) + 2
        return(piecols[dges])
    }
    else if (v %in% bad) {
        return("white")
    }
    else {
        return("black")
    }
})(V(IFNgraph)$name)))
V(IFNgraph)$pie.border = unname(as.list(Vectorize(function(v) {
    if (v %in% rownames(posthoc)) {
        dges = as.vector(posthoc[v, COIs]) + 2
        return(piecols[dges])
    }
    else if (v %in% bad) {
        return("black")
    }
    else {
        return("black")
    }
})(V(IFNgraph)$name)))
V(IFNgraph)$frame.lty = 3
V(IFNgraph)$frame.color = NA
V(IFNgraph)$avexp = top.table[match(V(IFNgraph)$name, rownames(top.table)), ]$AveExpr
V(IFNgraph)$avexp = as.numeric(ifelse(is.na(V(IFNgraph)$avexp), max(V(IFNgraph)$avexp, na.rm = T), V(IFNgraph)$avexp))
avexppal <- c("lightgoldenrod3", "azure4", "black")
V(IFNgraph)$frame.color = avexppal[as.numeric(cut(V(IFNgraph)$avexp, breaks = quantile(top.table$AveExpr, probs = c(0, 0.25,
    0.75, 1)), include.lowest = T, right = T))]
E(IFNgraph)$width = 0.6
V(IFNgraph)$frame.width = 10


par(mfrow = c(1, 1), mar = c(0, 0, 0, 0), family = "bold")

graph2plot = delete.edges(IFNgraph, which(E(IFNgraph)$color == "white"))
graph2plot = delete.vertices(graph2plot, which(degree(graph2plot) == 0))
V(graph2plot)$label = V(graph2plot)$name
V(graph2plot)$label.font = 2


E(graph2plot)$lty = 1


extrintr = sapply(E(graph2plot)$EXTRACELLULAR_INPUT, function(v) (sum(unlist(v))))

E(graph2plot)$lty = ifelse(extrintr > 0, 2, 1)

E(graph2plot)$width = ifelse(extrintr > 0, 0.9, 0.5)

E(graph2plot)$color = c("steelblue1", "gray87", "hotpink1", "purple3")[as.numeric(factor(E(graph2plot)$eloc))]


set.seed(1)


E(graph2plot)$weight = (1/(0.1 + 100 * extrintr))^2


l <- qgraph::qgraph.layout.fruchtermanreingold(get.edgelist(graph2plot, names = F), vcount = vcount(graph2plot), niter = 10000,
    area = 10 * (vcount(graph2plot)^2.4), repulse.rad = (vcount(graph2plot)^2.4), weights = (1/(0.1 + 100 * extrintr))^2)


V(graph2plot)$label.color = "black"

V(graph2plot)[unique(unlist(neighborhood(graph2plot, order = 1, mode = "all", nodes = c("CD274", "PDCD1", "CTLA4"))))]$label.color = "red3"

E(graph2plot)$arrow.size = 0.2
E(graph2plot)$arrow.width = 0.8

E(graph2plot)$color = adjustcolor(c("steelblue2", "gray85", "hotpink1", "purple3")[as.numeric(factor(E(graph2plot)$eloc))],
    alpha.f = 0.65)

plot(graph2plot, layout = l, vertex.frame.width = 10, vertex.label.cex = 0.42, edge.lty = E(graph2plot)$lty, vertex.label.degree = -pi/2,
    vertex.color = "white", vertex.frame.lty = "solid")
```

# 7 Expression Correlation between DEGs and Cell Markers

```
irDEGs = unique(c(Reduce(intersect, list(names(which(apply(posthoc[, c(1)], 1, sum) <= 0)), names(which(apply(posthoc[, c(2)],
    1, sum) <= 0)), names(which(apply(posthoc[, c(5:6)], 1, sum) > 1)))), Reduce(intersect, list(names(which(apply(posthoc[,
    c(1)], 1, sum) >= 0)), names(which(apply(posthoc[, c(2)], 1, sum) >= 0)), names(which(apply(posthoc[, c(5:6)], 1, sum) <
    -1))))))

mat = removeBatchEffect(d0, batch = conf, batch2 = conf2)


GOIs = c(irDEGs, "FOXP3")


BC = c("CD19", "MS4A1", "CD22", "CD79A", "CD79B", "TNFRSF17")

TC = c("CD3D", "CD3E", "CD3G", "CD4", "CD8A", "CD8B")

APC = c("CD68", "CD163", "CCL13", "CD209")


NK = c("ZNF205", "IL21R", "GZMB", "GTF3C1")


unigenes = unique(c(GOIs, BC, TC, APC, NK))


aecdf = Vectorize(ecdf(top.table$AveExpr))


genann = data.frame(row.names = unigenes, irDEG = factor(unigenes %in% GOIs), `B Cell Marker` = factor(unigenes %in% BC),
    `T Cell Marker` = factor(unigenes %in% TC), `NK Cell Marker` = factor(unigenes %in% NK), `APC Marker` = factor(unigenes %in%
        APC), `Avg. Expr. Percentile` = cut(100 * round(aecdf(top.table[unigenes, ]$AveExpr), 2), breaks = c(0, 25, 75, 100),
        include.lowest = T, right = F), check.names = F)

genann$irDEG = as.numeric(factor(as.numeric(posthoc[match(rownames(genann), rownames(posthoc)), 5, drop = T])))

anncolors = lapply(genann, function(i) {

    if (length(levels(i)) == 2) {
        return(setNames(c("white", "black"), levels(i)))
    }
    else {
        if (is.numeric(i)) {
            return(setNames(c("turquoise2", "white", "maroon2"), factor(unique(sort(genann$irDEG - 2)))))
        }
        else {
            return(setNames(RColorBrewer::brewer.pal(length(levels(i)), "YlGn"), levels(i)))
        }
    }
})
genann$irDEG = factor(genann$irDEG - 2)


mat = removeBatchEffect(d0, batch = conf, batch2 = conf2)

mat = mat[match(unigenes, rownames(mat)), ]


GxGcors = combn(as.list(as.data.frame(t(mat))), 2, function(l) {
    xc = cor.test(l[[1]], l[[2]], alternative = "two.sided", method = "spearman", continuity = T)
    return(data.frame(Rho = xc$estimate, P = xc$p.value))
}, simplify = F)

GxGnames = combn(rownames(mat), 2, function(l) (data.frame(G1 = l[[1]], G2 = l[[2]])), simplify = F)

GxGcordf = cbind(dplyr::bind_rows(GxGnames), dplyr::bind_rows(GxGcors))

GxGcordf$Padj = p.adjust(GxGcordf$P)

GxGcordf$signif = ifelse(GxGcordf$Padj <= 0.05, "＊", "")

GxGcordf2tab = GxGcordf[GxGcordf$Padj <= 0.05, ]

GxGcordf2tab[, sapply(GxGcordf2tab, is.numeric)] = apply(GxGcordf2tab[, sapply(GxGcordf2tab, is.numeric)], 2, format, digits = 2)

DT::datatable(as.data.frame(GxGcordf2tab), caption = "Significant expression correlations (5% FDR)", rownames = F, escape = F,
    filter = "top", extensions = c("FixedColumns", "FixedHeader"), options = list(scrollX = F, paging = T, fixedHeader = T,
        lengthMenu = list(c(10, 30, 50, -1), c("10", "30", "50", "All"))))
```

```
addWorksheet(wb, "Expression correlations", gridLines = T)
writeDataTable(wb, "Expression correlations", as.data.frame(GxGcordf2tab), rowNames = T)

GxGcorvals = reshape2::acast(GxGcordf[, c("G1", "G2", "Rho")], G1 ~ G2, value.var = "Rho")


GxGcormat = matrix(data = NA, nrow = length(unigenes), ncol = length(unigenes), dimnames = list(unigenes, unigenes))

GxGcormat[rownames(GxGcorvals), colnames(GxGcorvals)] <- GxGcorvals

GxGcormat[lower.tri(GxGcormat)] <- t(GxGcormat)[lower.tri(GxGcormat)]


GxGcorvals = reshape2::acast(GxGcordf[, c("G1", "G2", "signif")], G1 ~ G2, value.var = "signif")


GxGcorsig = matrix(data = NA, nrow = length(unigenes), ncol = length(unigenes), dimnames = list(unigenes, unigenes))

GxGcorsig[rownames(GxGcorvals), colnames(GxGcorvals)] <- GxGcorvals

GxGcorsig[lower.tri(GxGcorsig)] <- t(GxGcorsig)[lower.tri(GxGcorsig)]


diag(GxGcormat) = 1
diag(GxGcorsig) = ""

dr = dist(GxGcormat, method = "euclidean")
clust_row = hclust(dr, method = "complete")

clust_row = as.hclust(reorder(as.dendrogram(clust_row), wts = match(rownames(mat)[with(genann, order(colnames(genann)))],
    clust_row$labels)))

paletteLength <- 8
myColor <- colorRampPalette(c("mediumpurple2", "white", "sienna2"))(paletteLength)
myBreaks <- seq(-1, 1, length.out = paletteLength + 1)

legend_labels = c(myBreaks[-length(myBreaks)], "Spearman Correlation")
legend_breaks = seq(-0.99, 0.99, length.out = paletteLength + 1)


pheatmap::pheatmap(GxGcormat, fontsize = 6.5, fontsize_number = 7, cutree_rows = 5, cutree_cols = 5, legend = T, annotation_legend = T,
    display_numbers = GxGcorsig, cluster_rows = clust_row, cluster_cols = clust_row, annotation_row = genann, color = myColor,
    border_color = "gray70", breaks = myBreaks, legend_labels = legend_labels, annotation_col = genann, annotation_colors = anncolors,
    treeheight_col = 15, treeheight_row = 15, angle_col = 90)
```

# 8 Comparison of irDGE results to other studies

## 8.1 Using dataset GSE220915

- Status Public on Feb 08, 2023
  Title Coordinated local overexpression of complement induced by interferon gamma in myositis
- Organism Homo sapiens
- Experiment type Expression profiling by high throughput sequencing

  - Summary Complement proteins are deposited in the muscles of patients with myositis. However, the local expression and regulation of complement genes within myositis muscle have not been well characterized. In this study, bulk RNA sequencing (RNAseq) analyses of muscle biopsy specimens revealed that complement genes are locally overexpressed and correlate with markers of myositis disease activity, including the expression of interferon-gamma (IFN𝛾)-induced genes. Single cell and single nuclei RNAseq analyses showed that most local expression of complement genes occurs in macrophages, fibroblasts, and satellite cells, with each cell type expressing different sets of complement genes. Biopsies from immune-mediated necrotizing myopathy patients, who have the lowest levels of IFN𝛾-induced genes, also had the lowest complement gene expression levels. Furthermore, data from cultured human cells showed that IFN𝛾 upregulates complement expression in macrophages, fibroblasts, and muscle cells. Taken together, our results suggest that in myositis muscle, IFN𝛾 coordinates the local overexpression of complement genes that occurs in several cell types.
- Overall design Gene expression profiling analysis of RNA-seq data for patients with different types of myositis (DM, AS, IMNM, IBM) and histologically normal muscle biopsies (NT).
- Contributor(s) Mammen AL, Pinal-Fernandez I
- Citation(s)

  Casal-Dominguez M, Pinal-Fernandez I, Pak K, Muñoz-Braceras S et al. Coordinated local RNA overexpression of complement induced by interferon gamma in myositis. Sci Rep 2023 Feb 4;13(1):2038. PMID: 36739295
  Abad C, Pinal-Fernandez I, Guillou C, Bourdenet G et al. IFNγ causes mitochondrial dysfunction and oxidative stress in myositis. Nat Commun 2024 Jun 26;15(1):5403. PMID: 38926363
  Najjar R, Alessi H, Pinal-Fernandez I, Mammen AL et al. Distinct Transcript-Level Expression Profiles and Unique Alternative Splicing in Inflammatory Myopathies. ACR Open Rheumatol 2024 Oct;6(10):690-699. PMID: 39073022

```
gsemat <- getGEO("GSE220915", GSEMatrix = T)

GSE220915_anonym_counts_hg38.tsv <- read.delim("~/mount-srv1/musellla/PhD_Projects/MelAutim-Colitis-FACS-NanoString/DATA/Myositis/GSE220915_anonym_counts_hg38.tsv.gz")

GSE220915_anonym_counts_hg38.ts = tibble::column_to_rownames(GSE220915_anonym_counts_hg38.tsv, "gene_id")


mart_export.txt <- read.delim("~/mount-srv1/musellla/PhD_Projects/MelAutim-Colitis-FACS-NanoString/DATA/Myositis/mart_export.txt.gz")


phenodata = pData(phenoData(gsemat[[1]]))

rownames(phenodata) = str_remove(phenodata$title, "_[a-z]+$")

ygse <- DGEList(counts = GSE220915_anonym_counts_hg38.ts, group = make.names(phenodata$`diagnosis:ch1`), samples = phenodata,
    genes = mart_export.txt[match(str_remove_all(rownames(GSE220915_anonym_counts_hg38.ts), "\\.[0-9]+$"), mart_export.txt$Gene.stable.ID),
        ])


ygse = calcNormFactors(ygse, method = "none")

fbe = edgeR::filterByExpr(ygse, group = ygse$samples$group, min.count = 10)


print("genes that meeet the minimum-count criterion in GSE220915:")
```

```
# [1] "genes that meeet the minimum-count criterion in GSE220915:"
```

```
table(fbe)
```

```
# fbe
# FALSE  TRUE 
# 39097 22017
```

```
print("genes analyzed in this study that do not meeet the minimum-count criterion in GSE220915:")
```

```
# [1] "genes analyzed in this study that do not meeet the minimum-count criterion in GSE220915:"
```

```
intersect(ygse$genes$Gene.name[match(names(fbe[!fbe]), rownames(ygse))], rownames(top.table_irmyo))
```

```
#  [1] "CCL17"    "IL26"     "CR2"      "CXCL6"    "CCL25"    "S100A7"   "CD1A"     "IL24"     "IFNB1"    "IFNL1"   
# [11] "PMCH"     "IL1RAPL2" "CLEC4C"   "GAGE1"    "KLRC2"    "CLEC6A"   "CTAGE1"   "IFNA7"    "IFNA17"   "CT45A1"  
# [21] "TPTE"
```

```
voomgse = edgeR::voomLmFit(calcNormFactors(ygse[fbe, ], method = "TMM"), design = model.matrix(~0 + group, data = ygse$samples),
    normalize.method = "none", plot = T)
```

```
pcagse = prcomp(t(voomgse$EList$E))
pca.p.raw = autoplot(pcagse, data = ygse$samples, colour = "group", size = 2.5, label = F) + ggtitle(sprintf("PCA on voom-transformed log-CPM  (n=%i)",
    nrow(ygse$samples))) + theme_minimal() + scale_shape_manual(values = c(16, 15)) + theme(legend.title = element_text(color = "black",
    face = "bold"), title = element_text(color = "black", face = "bold"), strip.text = element_text(size = 8), legend.text = element_text(size = 12)) +
    ggConvexHull::geom_convexhull(aes(fill = group), alpha = 0.25) + scale_fill_manual(values = viridis::turbo(n = length(unique(ygse$samples$group)))) +
    scale_color_manual(values = viridis::turbo(n = length(unique(ygse$samples$group))))

pca.p.raw
```

```
fit = voomgse
contrlev = paste0("group", rev(levels(ygse$samples$group)))
contrs = combn(colnames(coef(fit))[grepl("group", colnames(coef(fit)))], 2)
contrs = apply(contrs, 2, function(v) {
    ord = match(v, contrlev)
    if (ord[1] < ord[2]) {
        return(v)
    }
    else {
        return(v[c(2, 1)])
    }
})

print(matrix(t(contrs[c(2, 1), ]), ncol = nrow(contrs), nrow = ncol(contrs), dimnames = list(seq(1, ncol(contrs)), c("Case",
    "Reference"))))
```

```
#    Case                   Reference             
# 1  "groupmyositis..AS."   "groupmyositis..DM."  
# 2  "groupmyositis..AS."   "groupmyositis..IBM." 
# 3  "groupmyositis..AS."   "groupmyositis..IMNM."
# 4  "groupmyositis..AS."   "groupnormal.muscle"  
# 5  "groupmyositis..DM."   "groupmyositis..IBM." 
# 6  "groupmyositis..DM."   "groupmyositis..IMNM."
# 7  "groupmyositis..DM."   "groupnormal.muscle"  
# 8  "groupmyositis..IBM."  "groupmyositis..IMNM."
# 9  "groupmyositis..IBM."  "groupnormal.muscle"  
# 10 "groupmyositis..IMNM." "groupnormal.muscle"
```

```
contrs = paste(apply(t(contrs[c(2, 1), ])[grep("normal", t(contrs[c(2, 1), ])[, 2, drop = T]), ], 1, paste, collapse = " - ",
    sep = " "), collapse = ", ")
comptag = strsplit(contrs, ", ", perl = T)[[1]]
comptag = str_remove_all(comptag, "group")
contrs = strsplit(contrs, ", ", perl = T)[[1]]
contrs = paste(contrs, collapse = ", ")
command = paste("makeContrasts(", contrs, ",levels=model.matrix(~0+group,data = ygse$samples))", sep = "")
contr = eval(parse(text = command))


tmp <- contrasts.fit(fit, contr)
tmp <- eBayes(tmp, trend = F, robust = F)
plotSA(tmp)
```

```
top.table <- topTable(tmp, sort.by = "F", n = Inf)

colnames(top.table)[match(make.names(comptag), str_remove_all(colnames(top.table), "group"))] = comptag
posthoc = as.matrix(decideTests(tmp, method = "hierarchical", adjust.method = "BH", p.value = 0.05))
print(paste("how many significantly DE genes (F statistics, 5% FDR)?", length(which(top.table$adj.P.Val < 0.05))))
```

```
# [1] "how many significantly DE genes (F statistics, 5% FDR)? 14046"
```

```
print(paste("how many significant, pairwise tests (post-hoc moderated t-test, 5% FDR)?", sum(abs(posthoc))))
```

```
# [1] "how many significant, pairwise tests (post-hoc moderated t-test, 5% FDR)? 34887"
```

```
top.table$Gene.name = str_replace_all(top.table$Gene.name, "RIGI", "DDX58")

posttop.table = top.table
posttop.table[, match(str_remove_all(colnames(posthoc), "group"), colnames(posttop.table))] = posttop.table[, match(str_remove_all(colnames(posthoc),
    "group"), colnames(posttop.table))] * abs(posthoc)[rownames(posttop.table), ]


DT::datatable(as.data.frame(posttop.table[match(rownames(top.table_irmyo), posttop.table$Gene.name, nomatch = 0), ]), caption = "DGE results from GSE220915 of genes also analyzed in this study (post hoc 5% FDR correction)",
    rownames = T, escape = F, extensions = c("FixedColumns", "FixedHeader"), options = list(scrollX = F, paging = T, fixedHeader = T))
```

```
addWorksheet(wb, "DGE results from GSE220915", gridLines = T)
writeDataTable(wb, "DGE results from GSE220915", as.data.frame(posttop.table[match(rownames(top.table_irmyo), posttop.table$Gene.name,
    nomatch = 0), ]), rowNames = T)


irDEGs_thisstudy = top.table_irmyo[match(sort(c(irDEGs)), rownames(top.table_irmyo)), ]

irDEGs_GSE = top.table[match(irDEGs_thisstudy$Name, top.table$Gene.name), ]


checkgs = intersect(top.table$Gene.name, top.table_irmyo$Name)

ggpubr::ggscatter(data = data.frame(X = top.table_irmyo$AveExpr[match(checkgs, top.table_irmyo$Name)], Y = top.table$AveExpr[match(checkgs,
    top.table$Gene.name)], check.names = ), x = "X", y = "Y", color = "gray80", alpha = 0.5, add = "reg.line", add.params = list(color = "blue",
    fill = "lightgray"), conf.int = TRUE) + stat_cor(method = "pearson", label.x = -3, label.y = 12) + theme_minimal() +
    xlab("Avg. Expr. - This Study") + ylab("Avg. Expr. - GSE220915") + xlim(c(-5, 20)) + ylim(c(-5, 20))
```

```
mergemat = cbind(irDEGs_thisstudy[, grep("\\.{3,3}", colnames(irDEGs_thisstudy))], irDEGs_GSE[, grep("\\s-\\s", colnames(irDEGs_GSE))])

mergemat = mergemat[complete.cases(mergemat), ]

colnames(mergemat) = str_remove_all(colnames(mergemat), "group")

colnames(mergemat) = str_replace_all(colnames(mergemat), "\\.{3,3}", " - ")

colnames(mergemat) = str_replace_all(colnames(mergemat), "\\.{2,2}([A-Z]+)\\.", " (\\1)")

colnames(mergemat) = str_replace_all(colnames(mergemat), "\\.", " ")

colnames(mergemat) = tools::toTitleCase(colnames(mergemat))

colnames(mergemat) = str_replace_all(colnames(mergemat), "\\(as\\)", "(AS)")

paletteLength <- 8


myBreaks <- c(seq(min(mergemat), 0, length.out = ceiling(paletteLength/2) + 1), seq(max(mergemat)/paletteLength, max(mergemat),
    length.out = floor(paletteLength/2)))

annrow = data.frame(Dataset = ifelse(grepl("^ir|NDC|TIF|Mi2", colnames(mergemat)), "This Study", "GSE220915"), row.names = colnames(mergemat))


aecdf = Vectorize(ecdf(top.table_irmyo$AveExpr))
becdf = Vectorize(ecdf(top.table$AveExpr))


genann = data.frame(row.names = sort(c(irDEGs)), `Avg. Expr. Prc. - This Study` = cut(100 * round(aecdf(top.table_irmyo[sort(c(irDEGs)),
    ]$AveExpr), 2), breaks = seq(0, 100, by = 10), include.lowest = T, right = F), `Avg. Expr. Prc. - GSE220915` = cut(100 *
    round(becdf(top.table[match(irDEGs_thisstudy$Name, top.table$Gene.name), ]$AveExpr), 2), breaks = seq(0, 100, by = 10),
    include.lowest = T, right = F), check.names = F)


anncol = data.frame(Dataset = ifelse(grepl("^ir|NDC|TIF|Mi2", colnames(mergemat)), "This Study", "GSE220915"), row.names = colnames(mergemat))

anncolors = list(Dataset = setNames(c("gray45", "gray90"), c("This Study", "GSE220915")), `Avg. Expr. Prc. - This Study` = setNames(c("white",
    colorspace::darken(RColorBrewer::brewer.pal(length(seq(10, 100, length.out = 9)), "BuPu"))), levels(genann$`Avg. Expr. Prc. - This Study`)),
    `Avg. Expr. Prc. - GSE220915` = setNames(c("white", colorspace::darken(RColorBrewer::brewer.pal(length(seq(10, 100, length.out = 9)),
        "BuPu"))), levels(genann$`Avg. Expr. Prc. - This Study`)))

myColor <- colorRampPalette(c("dodgerblue2", "white", "violetred2"))(8)

pheatmap::pheatmap(t(mergemat[, !grepl("TIF1.+Mi2", colnames(mergemat))]), breaks = sort(unique(c(seq(min(mergemat), 0, length.out = 5),
    seq(0, max(mergemat), length.out = 5)))), color = myColor, annotation_names_row = T, annotation_legend = T, border_color = "black",
    angle_col = 315, annotation_row = annrow, annotation_col = genann, annotation_colors = anncolors, treeheight_col = 10,
    treeheight_row = 20, legend_labels = c(signif(sort(unique(c(seq(min(mergemat), 0, length.out = 5), setdiff(seq(0, max(mergemat),
        length.out = 5), max(mergemat))))), 2), "log2 FC"), legend_breaks = sort(unique(c(seq(min(mergemat), 0, length.out = 5),
        seq(0, max(mergemat), length.out = 5)))), fontsize_row = 8, fontsize = 8, fontsize_col = 8, clustering_distance_cols = "euclidean",
    clustering_distance_rows = "euclidean", clustering_method = "ward.D2")
```

## 8.2 Using literature mining (ENQUIRE)

Expanding Networks by Querying Unexpectedly Inter-Related Entities (ENQUIRE) is a text-mining and network reconstruction method aimed at supporting biomedical literature review and facilitating biomedical information retrieval. It’s described in journal.pcbi.1012745. The software can be downloaded at https://doi.org/10.6084/m9.figshare.29357207.v1. It’s necessary to install apptainer beforehand. Further information on its usage is available on GitHub. Skip to step *D* if you are using the provided graph databased available as supplementary files.

A - Firstly, A PubMed query must be formulated and PMIDs saved to a .txt file. We formulated the following queries:

1. Research papers with a focus on immune-related adverse events (irAEs) leading to autoimmune coniditions (“10 years” and “Abstract” filters, 239 results as of 17.06.2025);

```
((("immune checkpoint inhibitor*"[MeSH Terms])) AND ("autoimmune diseases"[MeSH Terms])) NOT ("review"[Publication Type])
```

2. Research papers with a focus on either ICI therapy for melanoma (the majority of irMyositis patients in this study had a cutaneous melanoma diagnosis) or immunological aspects of dermatomyositis (“10 years” and “Abstract” filters, 660 results as of 17.06.2025).

```
(("immune checkpoint inhibitors/adverse effects"[MeSH Terms] AND "melanoma"[MeSH Terms]) OR ("dermatomyositis/immunology"[MeSH Terms])) NOT ("review"[Publication Type])
```

B - After saving the results to a `input.txt` file, we run ENQUIRE using the latest `apptainer image` (SIF) available at figshare: . With the above inputs, the script could take several hours on systems with less than 20 CPU cores.

```
# increase number of cores j if possible
# -r 100 to functionally disable network expansion
./ENQUIRE-Neo4j.sif ENQUIRE.sh -t study_name -i input.txt -j 4 -r 100
```

C - The output will be created at `tmp-study_name` in the current working directory. To construct a graph database from ENQUIRE output, we use neo4j (already installed within the SIF image). First, choose a and create `JOBDIR` directory path under which the graph database will be created

```
export JOBDIR=$(pwd)/your_directory_name_here

mkdir -p $JOBDIR/var/lib/neo4j/{plugins,data,run}
mkdir -p $JOBDIR/var/log/neo4j
mkdir -p $JOBDIR/output
```

Then, construct the database as follows

```
apptainer run \
  --mount dst=/var/lib/neo4j,src=$JOBDIR/var/lib/neo4j \
  --mount dst=/var/log/neo4j,src=$JOBDIR/var/log/neo4j \
  ENQUIRE-Neo4j.sif make_graphDB_V4.py --inputfolder tmp-study_name/study_name/ --db-name neo4j --outputfolder $JOBDIR/output
```

This command should have generated all necessary files for constructing a neo4j graph database.

D - Activate the graph database via the following command:

```
apptainer exec \
--mount dst=/var/lib/neo4j,src=$JOBDIR/var/lib/neo4j \
--mount dst=/var/log/neo4j,src=$JOBDIR/var/log/neo4j \
ENQUIRE-Neo4j.sif neo4j console
```

Neo4j is now available at http://localhost:7474/ or as a remote DBMS using Neo4j Remote Desktop app (recommended) via URL neo4j://localhost:7687. Nodes are of three possible types: `Gene`, `MeSH`, and `Literature`. Relations are of two possible types: `CO_OCCURS` and `HAS_SOURCE`. If using the provided databases, define the `JOBDIR` variable as the folder name from one of `enquire_generated_ICI_AND_AID.tar.gz` or `enquire_generated_ICI-Melanoma_OR_DM.tar.gz` after extraction.

We used Neo4j/Cypher queries that retrieved PMIDs and MeSH associated to irDEGs to mine relevant literature to compare our results to previous studies. Some examples are provided below.

```
MATCH (m:MeSH)-[:CO_OCCURS]-(g:Gene)
MATCH (m)-[:HAS_SOURCE]-(l:Literature)-[:HAS_SOURCE]-(g)
WHERE any(x IN g.ENTITY WHERE x IN [
'IFNG',
'FOXP3'
]) AND m.ENTITY =~ '.*myositis.*|.*immune checkpoint.*|.*lymphocyte.*|.*natural killer.*'
//RETURN DISTINCT g.ENTITY AS irDEG, m.ENTITY AS MeSH,l.PMID AS PMID, l.`Link.explicit` AS LINK
RETURN m,l,g
```

Orange: genes. Aquamarine: MeSH. Red: PMID.

```
MATCH (m:MeSH)-[:CO_OCCURS]-(g:Gene)
WHERE any(x IN g.ENTITY WHERE x IN [
'BST2',
'CASP10',
'CCL14',
'CFB',
'CR1',
'DDX58',
'FCGR1A',
'FN1',
'IFI16',
'IFI27',
'IFI35',
'IFIH1',
'IFIT1',
'IFIT2',
'IFITM1',
'IFNG',
'IL1RAP',
'IRF7',
'ISG15',
'ISG20',
'KLRC2',
'MX1',
'NT5E',
'OAS3',
'STAT2',
'TGFB2',
'TIGIT',
'TNFRSF8',
'PDCD1',
'CD274',
'CTLA4',
'FOXP3'
]) AND m.ENTITY =~ '.*myositis.*|.*immune checkpoint.*|.*lymphocyte.*|.*natural killer.*'
RETURN g,m
```

Orange: genes. Aquamarine: MeSH.

# 9 Save Data to Excel File

```
saveWorkbook(wb, "myositis_analysis.xlsx", overwrite = T)
```
